# Comparing estimated cost‐effectiveness of micronutrient intervention programs using primary and secondary data: evidence from Cameroon

**DOI:** 10.1111/nyas.14726

**Published:** 2021-12-09

**Authors:** Katherine P. Adams, Hanqi Luo, Stephen A. Vosti, Justin Kagin, Ismael Ngnie‐Teta, Alex Ndjebayi, Jules Guintang Assiene, Reina Engle‐Stone

**Affiliations:** ^1^ Institute for Global Nutrition Department of Nutrition University of California Davis Davis California; ^2^ Hubert Department of Global Health Rollins School of Public Health Emory University Atlanta Georgia; ^3^ Department of Agricultural and Resource Economics University of California Davis Davis California; ^4^ Kagin's Consulting Vacaville California; ^5^ Helen Keller International Yaoundé Cameroon

**Keywords:** micronutrient interventions, household consumption and expenditure survey, cost‐effectiveness, 24‐h dietary recall, Cameroon

## Abstract

Designing a cost‐effective portfolio of micronutrient intervention programs is complex and generally undertaken with limited data. We developed the MINIMOD‐Secondary Data (MINIMOD‐SD) tool, which uses household consumption and expenditure survey data and other secondary data to estimate apparent nutrient intakes and model the effectiveness and cost‐effectiveness of micronutrient intervention programs. We present the SD tool methodology and results in the context of Cameroon, with a particular focus on vitamin A (VA) for children and folate for women of reproductive age (WRA). We compared the MINIMOD‐SD tool estimates with those of the full MINIMOD tool, which uses 24‐h dietary recall data. The SD tool consistently underestimated folate intake among women (median (IQR): 230 (143,352) versus 303 (244,367) μg dietary folate equivalents (DFEs)/day) and especially VA among children (141 (64,279) versus 227 (102,369)). Qualitatively, however, the two tools were generally consistent in predicted subnational patterns of micronutrient adequacy and identification of effective and cost‐effective (cost per child/WRA moving from inadequate to adequate intake) interventions. Secondary data and the MINIMOD‐SD tool can provide policymakers with information to qualitatively assess deficiency risks and identify cost‐effective interventions. However, accurately quantifying individual‐level deficiency or dietary inadequacy and intervention effectiveness and cost‐effectiveness will likely require individual‐level dietary data and biomarker measurements.

## Graphical abstract

We developed the MINIMOD‐Secondary Data (MINIMOD‐SD) tool, which uses household consumption and expenditure survey data and other secondary data to estimate apparent nutrient intakes and model the effectiveness and cost‐effectiveness of micronutrient intervention programs. We present the SD tool methodology and results in the context of Cameroon, with a particular focus on vitamin A (VA) for children and folate for women of reproductive age. We compared the MINIMOD‐SD tool estimates with those of the full MINIMOD tool, which uses 24‐h dietary recall data. Qualitatively, the two tools were generally consistent in predicted subnational patterns of micronutrient adequacy and identification of effective and cost‐effective interventions.

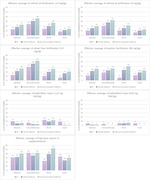

## Introduction

Micronutrient deficiencies, which are prevalent and particularly consequential among young children and women of reproductive age (WRA) across many low‐ and middle‐income countries (LMICs), contribute substantially to the global burden of disease and excess mortality.^1^, ^2^ Excess mortality coupled with the detrimental long‐term effects of undernutrition on health, cognitive development, human capital acquisition, work capacity, and earnings potential have substantial private and social costs.^3^ Despite the associated adverse health effects and high costs, micronutrient deficiencies remain a major public health challenge;^4^ worldwide, an estimated 2 billion people are deficient in one or more micronutrients.^2^

In the long term, sustainably addressing micronutrient deficiencies and other forms of undernutrition should be made through nutrition‐focused improvements in food systems and changes in dietary patterns alongside economic development policies to stimulate economic growth and help alleviate poverty. Increasing micronutrient intakes and decreasing the negative impacts of deficiencies today, however, require solutions that can be implemented and brought to scale in the more immediate term. A range of such interventions exist, including supplementation, fortification, and biofortification.^2^, ^5^ However, prioritizing, financing, and managing an effective and cost‐effective portfolio of national and targeted subnational micronutrient intervention programs is a complex task that is often done without adequate data by various public and private sector entities.^5^, ^6^ To help manage this complexity and provide a systematic framework for developing a coordinated micronutrient intervention strategy, the Micronutrient Intervention Modeling (MINIMOD) project has developed a set of tools to provide policymakers with data‐driven, spatially and temporally explicit information on the benefits, costs, relative cost‐effectiveness, and economically optimal sets of micronutrient intervention programs.

The full MINIMOD tool, described elsewhere in detail,^6^ has three component models. The nutrition benefits model uses nationally representative 24‐h dietary recall (24HR) and biomarker data to estimate the spatial distribution of micronutrient deficiencies among target population groups and, combined with the estimates of program reach, predicts the spatially and temporally explicit impacts of all combinations of alternative micronutrient intervention programs.^7^ The cost model uses budgetary data, known unit costs, and expert knowledge to estimate spatially and temporally explicit costs of alternative micronutrient intervention programs and combinations of interventions.^8^ Finally, the economic optimization model combines predicted nutrition benefits with estimated intervention program costs in a constrained optimization framework and uses linear programming to identify an economically efficient portfolio of interventions that are, again, explicit over time and space.^9^

The full MINIMOD tool has been developed and used to identify cost‐effective intervention programs for a range of micronutrients in Cameroon and Ethiopia, and these results have helped shape the direction of micronutrient intervention policies and programs in these countries.^10^, ^11^ However, unlike Cameroon and Ethiopia, most LMICs do not have recent, nationally representative, individual‐level dietary intake or biomarker data.^12^, ^13^, ^14^ Without undertaking a national survey of individual dietary intake, which is often perceived as time‐consuming and prohibitively costly,^13^ most LMICs are precluded from using the full MINIMOD tool to help more efficiently design, target, and manage their micronutrient intervention programs. To overcome the scarcity of nationally representative dietary intake data, we developed the MINIMOD‐secondary data (or MINIMOD‐SD) tool, which primarily utilizes secondary data that are regularly collected in most LMICs to estimate the benefits, costs, and cost‐effectiveness of micronutrient intervention programs.

Recognizing that conducting nationally representative dietary intake surveys via observed weighted food records or 24HR has proven infeasible and/or of low priority for most LMICs,^13^ many researchers have turned to secondary sources of data, including Food and Agricultural Organization Food Balance Sheets (FBS) (e.g., Refs. 15 and 16) and household consumption and expenditure surveys (HCESs) (e.g., Refs. 12, 17–19). While FBS data have the advantage of being collected annually for almost all countries, they are limited to providing information about food availability (rather than consumption) at the national level without the possibility of spatial (or other) disaggregation.^20^ HCES data, on the other hand, are regularly conducted, nationally and subnationally representative surveys that collect data on recent household acquisition and/or consumption of food.^20^, ^21^

The MINIMOD‐SD tool uses HCES data as proxies for individual‐level dietary intake data. Building on other recent efforts using HCES data to assess the need for and impacts of micronutrient interventions,^19^, ^22^, ^23^, ^24^, ^25^, ^26^ the MINIMD‐SD tool incorporates several key advances. In particular, we designed the tool to use HCES‐based estimates in combination with other secondary data sources (e.g., the Demographic and Health Survey data on supplementation coverage) to allow for consideration of a wider range of potential interventions than has typically been done. We also model the impacts, cost, and cost‐effectiveness of both individual and combined interventions at the national and subnational levels, which better simulates the complex decision space faced by policymakers compared with modeling single interventions and also allows for consideration of targeting at subnational levels. We also use several different metrics to measure the impact of alternative intervention programs. These varying measures of impact, defined in detail below, provide a broad assessment of how well a program is predicted to reach its target population but also allows for building a more nuanced picture of program performance by considering whether the program is reaching households or individuals who are at risk of deficiency and whether the program is predicted to provide sufficient quantities of micronutrient to overcome inadequacies. Finally, and importantly, we have benchmarks with which to compare both our estimates of the need for and nutrition benefits of micronutrient intervention programs as well as the estimated costs and cost‐effectiveness of those interventions, which allows us to identify where the MINIMOD‐SD tool approach does and does not perform well relative to the benchmark.

In this paper, we present the SD tool methodology for using secondary data to estimate the need for, and to predict the impacts, cost, and cost‐effectiveness of alternative micronutrient intervention programs at national and subnational levels over a 10‐year planning time horizon. In addition to presenting the SD tool methodology, our objective was to assess the reliability of the SD tool estimates for informing policy discussions by comparing the SD tool estimates with benchmark estimates generated via the full MINIMOD tool, which are based on nationally representative 24HR data and cost information gleaned from country‐specific budgets, known unit costs, and key informants/experts. Specifically, for the pilot country of Cameroon and focusing on vitamin A (VA) for children aged 6–59 months and folate for WRA, we evaluated the performance of the SD tool relative to the full tool on the basis of a set of qualitative questions relevant for informing discussions around micronutrient policies and programs. First, do the nutrition need estimates derived from the SD tool suggest the same problems (i.e., high levels of inadequate micronutrient intake) as the full tool, and are the spatial patterns of inadequate micronutrient intake similar between the two tools? To answer this question, we compared the MINIMOD‐SD and full tool estimates of baseline dietary micronutrient intake and the prevalence of inadequate micronutrient intake. Second, how similar are the national and subnational rankings of existing and potential micronutrient interventions based on the nutrition benefit estimates derived from each tool? Here, we compared the estimates of the consumption of fortifiable and biofortifiable foods and the predictions of the effective coverage of each intervention and select combinations of interventions. Finally, how similar are the estimates of the cost‐effectiveness of these interventions that emerge from each tool? To answer this question, we compared the relative rankings of predicted impacts, cost, and cost‐effectiveness of alternative micronutrient intervention programs. These comparisons provide insight into the types of policy questions that analyses of secondary data using the SD tool methodology may or may not be suited to inform. They also help identify supplemental primary data collection that might be undertaken to help overcome some of the systematic limitations inherent in the use of secondary data for the purposes of informing discussions around micronutrient policies and programs.

## Methods

### HCES and 24HR data

For the MINIMOD‐SD tool (the tool overview provided in the Supplementary Methods and Figs. S1 and S2, online only), we used HCES data from the 2007 National Household Survey (*n* = 11,391 households), the Third Cameroon Household Survey (Troisième Enquête Camerounaise auprès des Ménages, ECAM3), to estimate apparent food consumption over 10 days of recall. Following Fiedler and Lividini,^27^ we use the term *apparent* food consumption to emphasize that food consumption estimates were based on reported food acquisition and the assumptions that (1) all food acquired during the recall period was consumed during the recall period without waste or food loss, and (2), as described below, food was distributed to individual household members according to their age‐ and sex‐specific energy requirements. The ECAM3 was conducted by the Cameroon National Institute of Statistics from September through December of 2007 using a two‐stage stratified random sample design.^28^ Additional information on the ECAM3 sampling strategy and data collection is provided in the Supplementary Methods (Supporting Information, online only). The sample characteristics are described elsewhere.^29^

As detailed in Engle‐Stone *et al*.,^30^ the 24HR data used for the full MINIMOD tool were collected as part of a nationally representative, multistage, cluster dietary survey conducted from September to December, 2009. The survey, which included households (*n* = 1002) with at least one child aged 12–59 months and one WRA, was designed to be representative of the three geographically defined “macroregions”: the major metropolitan areas of Yaoundé and Douala, the North (composed of the Extreme North, North, and Adamawa regions), and the South (composed of the Northwest, Southwest, West, Littoral (excluding Douala), Centre (excluding Yaoundé), East, and South regions). Selected households from each of the 30 clusters per macroregion were visited twice. The first visit was used to obtain informed consent and administer the 24‐h interview among a randomly selected 10% of households. Each household was visited a second time 2 days later to administer the 24HR to the full sample.^31^ The characteristics of the households with members who participated in the 24HR are described elsewhere.^30^

Differences between the HCES and 24HR with regard to the sample populations and years during which data were collected suggest that the estimates of food consumption and micronutrient intake are not exactly comparable. However, because the ages of children and WRA in both samples were similar (the average age in the HCES sample versus 24‐h sample was 2.3 versus 2.5 years among children and 28.1 versus 27.1 years among WRA), both surveys were representative at the national and subnational levels, and since we would not expect significant changes in dietary patterns between 2007 and 2009, both data sources should reflect population diets and allow for credible comparisons in answering the policy questions identified above.

### Food consumption and dietary micronutrient intake

To estimate apparent food consumption and micronutrient intake using the ECAM3 data, we began by converting reported quantities of food acquired by household during the recall period to standard units. Because quantities of food were reported in a wide range of units, many of which were nonstandard units (e.g., heap, bowl, and sack), and we did not have conversion factors for a majority of the (food‐specific) nonstandard units, we imputed a price‐per‐gram estimate for each food, which we then used to convert all food acquisitions to grams.^32^, ^33^ Specifically, using the total amount paid for the acquisition of each food as reported in the ECAM3 data, we calculated the median price‐per‐gram for all food acquisitions reported in standard units and then divided the total reported amount spent (or value) by the median price to estimate the total quantity acquired in grams. Where applicable, the total quantity was adjusted for the edible portion and the yield factor from cooking. No adjustments were made for waste or food loss, and it was assumed that all food acquired by the household was consumed by household members during the recall period. The daily average of the total quantity of each food acquired during the 10 days of recall was used as a proxy for usual household consumption.

Each food item was then matched with a food composition table entry to estimate the total apparent household energy and nutrient intake from each food. Foods were primarily matched with entries from the Nutrition Coordinating Center (NCC) Nutrient Database for Standard Reference (NDSR),^34^ supplemented with entries from the West African Food Composition Table^35^ and the USDA Food Composition Database.^36^ In addition to specific food items, the ECAM3 food list also included an “other” category for each food group (e.g., other cereals, other fruits, etc.). Foods categorized as “other” were not specified, so we estimated the nutrient value of these foods by assigning the average nutrient values of all specific foods in each category, omitting extreme outliers (e.g., given its high relative caloric density, an avocado was omitted from the average nutrient value calculation for “other fruits”). For a small number of foods that were assumed to be consumed cooked but for which only raw nutrient composition values were available (e.g., eggplant), we applied nutrient retention factors^36^ to adjust for nutrient loss during cooking. On the basis of qualitative observation of recipes during the 24HR data collection effort, unrefined palm oil (which was listed separately from refined palm oil in the ECAM3 food list) in Cameroon is sometimes boiled during the cooking of a dish and sometimes added to a dish after cooking. Given that retention factors for red palm oil are ∼50% (though with variability depending on cooking duration and temperature)^37^ and the variation in use during and after cooking, for both the ECAM3 and 24HR data, we conservatively assumed the average retention rate of VA in unrefined palm oil was 50%.

The ECAM3 also included a small section on expenditures on foods consumed away from home (FAFH). However, because many of the items were aggregate or catch‐all FAFH categories (e.g., “canteen catering services” or “other dishes taken outside”) and because nearly all reported quantities of FAFH were reported in nonstandard units, we were unable to incorporate food consumption and nutrient intake from FAFH.

We used the adult male equivalent (AME) method^38^ to estimate individual apparent food consumption and apparent nutrient intake from the household‐level apparent consumption. The AME method assumes that intrahousehold food distribution is proportional to each household member's age‐ and sex‐specific energy requirements. Each household member was, therefore, assigned an AME weight, which was the ratio of household member's energy requirements given their age and sex to the energy requirements of a male age 18–30 years, assuming moderate physical activity levels.^39^ The quantity of each food apparently consumed by each household member (or, likewise, apparent nutrient intake) was then calculated as the total household apparent consumption (or the total household apparent nutrient intake) multiplied by the individual's AME weight. Because pregnant and lactating women were not identifiable in the ECAM3 data, our AME calculations for all WRA were based on energy requirements for nonpregnant, nonlactating WRA. Extreme outliers in apparent consumption of each food in the food list, defined as apparent consumption per AME per day above the 95th percentile of apparent consumption per AME per day, were replaced with the 95th percentile value.

For the full model analysis, we used the SIMPLE macro, a streamlined software that utilizes the underlying theory of the National Cancer Institute method for usual intake analysis, to estimate usual dietary intake and prevalence of inadequate intake of VA and folate.^40^ Similar to the ECAM3 analysis, food items were linked with food composition data from the NCC NDSR,^34^ supplemented with entries from other tables^35^, ^36^ and from the literature, where necessary. Estimates of nutrient intake from breast milk were included in the calculation of total energy and nutrient intake estimates for children who were reported to be breastfeeding (12.7%). To do so, estimates of average daily breast milk intake^41^ were combined with estimated energy and folate content^41^ (for a total of 357 kcal/day and 49 μg dietary folate equivalents (DFEs)/day, respectively), and with measured values of breast milk VA concentration from a national survey.^7^, ^10^ We extrapolated energy and nutrient intakes to approximate those for a 6‐ to 59‐month age range on the basis of the estimated prevalence of breastfeeding for different age ranges.^7^

We used the cut‐point method to classify the adequacy of baseline (i.e., from dietary sources only without fortification or supplementation) apparent intake by comparing baseline dietary apparent intake with the estimated average requirement (EAR), which is the appropriate dietary reference intake value for the assessment of the population‐level prevalence of inadequacy.^42^ That is, the prevalence of inadequate intake was estimated by classifying apparent intake below the EAR as inadequate and calculating the proportion of the sample below this threshold. Table S1 (online only) summarizes EAR values for VA for children aged 6–59 months and folate for WRA. Because EARs are not defined for children below 1 year of age, we used the EARs for age 1–3 years for children aged 6–12 months. Also note that because breastfeeding status was not captured in the ECAM3 data, for all children, HCES‐based dietary VA intake estimates did not account for potential intake from breastmilk. Given this, we compared the SD tool estimates with the full tool estimates on the basis of both the full sample of children in the 24HR data and to the subset of nonbreastfeeding children in the 24HR data.

### Micronutrient intervention programs and nutrition benefits

We modeled the impacts of five individual interventions (and select combinations) to deliver VA to children aged 6–59 months: fortified refined oil, wheat flour, bouillon, biofortified maize, and high‐dose VA supplementation (VAS). VAS and fortified refined oil are ongoing intervention programs in Cameroon, while the others are hypothetical (although some amount of bouillon is voluntarily fortified with VA^7^). For fortified oil, we modeled two scenarios: one based on the target fortification level as currently mandated in Cameroon (12 mg/kg), and another based on the actual average levels (9 mg/kg).^43^ We also modeled two scenarios for biofortified maize. The first scenario assumed all maize in the country was replaced with a biofortified variety that provided 1.25 μg retinol activity equivalents (RAE) of VA per gram of maize. While 100% replacement is unlikely, this scenario provided an upper bound on the potential impacts of maize biofortification in Cameroon. We also modeled as a scenario in which half of the maize in the country was replaced with the biofortified variety that provided 1.25 μg RAE of VA per gram of maize, or, equivalently, the biofortified variety was assumed to provide an average of 0.63 μg RAE of VA per gram of maize.

We modeled the impacts of folic acid intervention programs (wheat flour and bouillon fortification) on the adequacy of intake among WRA (age 15–49 years). Cameroon mandates the fortification of wheat flour with folic acid (among other micronutrients) at 5 mg/kg, while the fortification of bouillon with folic acid is hypothetical. For food products containing some proportion of a food vehicle (e.g., wheat flour in bread or oil in beignets), we calculated fortifiable food equivalents by multiplying the quantity (apparently) consumed by the percentage of the food vehicle in the food product. The modeling assumptions for each micronutrient intervention program are summarized in Table S2 (online only).

We assessed each intervention program based on two measures of impact, or “nutrition benefits.”^6^ The first metric, reach, was defined as the percentage of individuals in the target population who received a micronutrient intervention in any amount. For VA‐fortified refined oil, for example, reach was the percentage of children aged 6–59 months who consumed or apparently consumed any fortifiable oil in any amount during the recall period. The second metric, effective coverage, was the percentage of individuals who were both at risk of deficiency owing to inadequate baseline dietary intake and who also received sufficient additional intake from an intervention or multiple interventions to be classified as having sufficient intake (i.e., effective coverage was calculated as the percentage point difference in the prevalence of inadequate intake in scenarios with and without a given intervention or set of interventions). As noted above, we predicted these measures of impact at both the national level and by macroregion (Yaoundé/Douala, North, and South), with macroregions corresponding to the three strata used in the clustered sampling design of the 24HR survey.^7^

In addition to the ECAM3 food acquisition data, we tapped several other secondary data sources to predict the impacts of each intervention program using the MINIMOD‐SD tool. Demographic estimates and projections of the total population and population size of each target group at the national level for the years 2020–2029 were obtained from DemProj, an open access demographic projection application in the Spectrum software.^44^ Macroregion population shares/weights were calculated on the basis of the macroregional share of the total population according to the most recently available census data for Cameroon. Region‐specific estimates of the percentage of women currently pregnant were obtained from the Cameroon 2011 DHS data and were used to estimate the total population of pregnant and nonpregnant WRA. For the full tool, we used population estimates provided by the Lives Saved Tool (LiST).

For each potential VA‐fortified food vehicle considered, the additional contribution to dietary VA intake was calculated as daily apparent consumption of the food vehicle (g/day) multiplied by the assumed level of fortification (μg/g). The additional contribution of biofortified maize considered was similarly calculated as daily apparent consumption multiplied by the assumed level of biofortification. High‐dose VAS was converted to a daily equivalent intake of 167 μg RAE/day as described in Engle‐Stone *et al*.^7^

Finally, the additional contribution of folic acid fortification to daily folate intake was calculated as daily apparent consumption of wheat flour and bouillon (g/day) multiplied by the assumed level of fortification (μg/g) converted to DFEs of intake (level of fortification divided by 0.6 to account for better absorption of folic acid than food folate).

### Costs and cost‐effectiveness

For each intervention program, for the MINIMOD‐SD tool, we developed 10‐year cost models to estimate start‐up and recurring costs faced by industry, government, and program beneficiaries associated with planning, implementing, and operating the intervention. For fortification and biofortification interventions, cost models were developed and estimated using an activity‐ and ingredient‐based approach. That is, the cost model structures were based on the set of activities required to plan, execute, and manage the intervention. Each activity was then populated with a series of inputs (or “ingredients”) that go into performing each activity and the estimated cost associated with each input. For high‐dose VAS, a modified activity‐based approach was used. In short, this modified approach followed the LiST costing methodology and involved estimating unit costs, by visit/contact, for the inputs associated with undertaking activities directly associated with the delivery of supplements, while additional facility‐level direct and indirect costs are estimated as a proportion of the cost associated with each outpatient visit (on the basis of country‐specific estimates made by the World Health Organization (WHO) and available at WHO‐CHOICE website (http://www.who.int/choice/cost‐effectiveness/en/)), while program‐level (i.e., above facility‐level) costs, including supervision, transportation, communications/outreach, overall program management, and so on, are each estimated as a percentage of total cost per visit/contact.

To the extent possible, cost estimates for each input (or unit costs) were derived from secondary data sources and from published and gray literature (where, e.g., unit costs estimated from a similar intervention in a similar setting were adapted to the Cameroon context). See Supplementary Methods and Tables S3–S5 (online only) for a more detailed description of the costing methodology for each intervention program, including the full set of costed activities for fortification and biofortification interventions, as well as additional information on secondary data sources for cost information. The full MINIMOD tool costing methodology and data sources, which included budgetary data, known unit costs, and input from local experts, were as described in Kagin *et al*.^8^

Finally, for each intervention and combination of interventions, we calculated cost‐effectiveness, at the national and macroregion levels, by dividing 10‐year total costs by 10‐year total nutrition benefits, where total nutrition benefits were the number of children/WRA effectively covered, calculated as the number of children/WRA in the target population multiplied by the estimated percent effectively covered.

## Results

### Baseline micronutrient intake and prevalence of inadequate intake

Both the SD and full MINIMOD tools predicted a relatively high prevalence of inadequate dietary VA intake among children aged 6–59 months, especially among nonbreastfed children (Table 1, top panel; see Table S6 for estimates of daily apparent energy intake, online only). The magnitude of the problem appeared more pronounced on the basis of the SD tool (∼75% inadequate compared with ∼49% and ∼62% based on the full tool among all children and nonbreastfed children, respectively), as estimates of apparent dietary VA intake among children were consistently lower on the basis of the SD tool than the full tool, especially in the North macroregion. However, both tools predicted the same spatial patterns, with the highest prevalence of inadequate intake in the North and the lowest prevalence in the South.

**Table 1:** Estimated baseline apparent*^a^* dietary vitamin A intake among children aged 6–59 months, the dietary folate intake among women of reproductive age, and the prevalence of inadequate apparent intakes

Vitamin A among children aged 6–59 months							
		Mean intake		Median intake		Inadequate intake	
Tool	Geography	μg RAE/day	SE	μg RAE/day	IQR	%	SE
SD*^b^*	National	199	3	141	(64,279)	75	1
	Yaoundé/Douala	222	7	192	(107,299)	71	2
	North	88	3	67	(29,120)	96	1
	South	273	6	216	(113,375)	60	1
Full (all children)*^c^*	National	271	14	227	(102,369)	49	4
	Yaoundé/Douala	247	21	186	(89,383)	57	5
	North	166	13	142	(69,253)	63	4
	South	375	26	320	(177,581)	34	8
Full (nonbreastfed children)*^d^*	National	220	16	166	(80,298)	62	5
	Yaoundé/Douala	188	20	153	(72,263)	67	6
	North	127	15	97	(53,168)	85	4
	South	309	28	258	(151,412)	42	10
Folate among women of reproductive age							
		Mean intake		Median intake		Inadequate intake	
Tool	Geography	μg DFE/day	SE	μg DFE/day	IQR	%	SE
SD*^b^*	National	266	3	230	(143,352)	72	1
	Yaoundé/Douala	244	4	217	(143,318)	77	1
	North	287	6	244	(143,395)	67	1
	South	263	3	229	(142,346)	73	1
Full*^e^*	National	303	12	303	(244,367)	71	5
	Yaoundé/Douala	249	16	260	(210,313)	85	10
	North	355	23	352	(294,416)	52	12
	South	291	14	290	(237,349)	79	7

a Because estimates from the MINIMOD‐SD tool were based on household consumption and expenditure survey data, the term *apparent* is used to emphasize that the SD tool estimates were based on reported food acquisition and the assumptions that all food acquired during the recall period was consumed during the recall period without waste or food loss and that food was distributed to individual household members according to the age‐ and sex‐specific energy requirements.

b Refers to estimates from the MINIMOD‐SD tool using household consumption and expenditure survey data.

c Refers to estimates from the full MINIMOD tool using 24HR data, estimated for the full sample of children aged 6–59 months.

d Refers to estimates from the full MINIMOD tool using 24HR data, estimated for the subsample of nonbreastfed children aged 6–59 months.

e Refers to estimates from the full MINIMOD tool using 24HR data.

Abbreviations: DFE, dietary folate equivalents; IQR, interquartile range; RAE, retinol activity equivalents; SE, standard error.

Both tools also predicted high prevalence rates (>70% nationally) of inadequate apparent dietary folate intake among WRA, nationally and in each of the three macroregions (bottom panel of Table 1). The spatial patterns of inadequate folate intake were similar between the two tools, with the highest percentage of WRA with inadequate apparent dietary folate intake in the cities of Yaoundé and Douala and lowest in the North. Quantitatively, the point estimates of the prevalence of inadequate folate intake among WRA derived from the SD tool were generally similar to the point estimates from the full tool (and were nearly identical at the national level), and the SD tool estimates were not consistently higher (or lower) than the full tool estimates by macroregion.

### Intervention program nutrition benefits

Estimates of reach and apparent consumption among consumers of modeled VA intervention programs are presented in Table 2 for children aged 6–59 months. Predictions of the reach of fortified refined oil and wheat flour and biofortified maize were higher on the basis of the SD tool compared with the full tool, while the SD tool estimates of apparent daily consumption among consumers of these foods were generally underestimated relative to the full tool (Table 2). The subnational patterns of predicted reach and apparent consumption were, however, generally in agreement between the two tools. Among the food‐based interventions, the predicted reach of fortified bouillon had the highest predicted reach on the basis of both tools nationally and in each macroregion, while the estimated apparent daily consumption of bouillon was slightly lower on the basis of the SD tool. Finally, estimates of the reach of high‐dose VAS were based on the same data source for both tools (an external monitoring report from the 2015 campaign^45^) and were, therefore, consistent between the two.

**Table 2:** Reach of vitamin A intervention programs and apparent*^a^* consumption of food vehicles among children aged 6–59 months

		Reach*^b^*		Apparent consumption (g/day) among consumers*c*	
Tool	Intervention	%	SE	Median	IQR
Fortified refined oil					
SD*^d^*	National	70	0.9	4	(1,11)
	Yaoundé/Douala	92	1.0	7	(2,14)
	North	76	1.6	5	(1,12)
	South	57	1.3	2	(1,8)
Full (all children)*^e^*	National	53	1.9	13	(8,19)
	Yaoundé/Douala	79	2.9	13	(8,19)
	North	56	2.9	12	(8,17)
	South	39	3.0	13	(8,19)
Full (nonbreastfed children)*^f^*	National	54	1.9	13	(8,20)
	Yaoundé/Douala	79	2.5	14	(8,20)
	North	57	3.3	13	(9,19)
	South	40	2.9	14	(8,20)
Fortified wheat flour					
SD*^d^*	National	63	0.9	10	(3,23)
	Yaoundé/Douala	97	0.7	22	(13,32)
	North	55	1.7	8	(3,22)
	South	55	1.3	5	(2,13)
Full (all children)*^e^*	National	48	1.9	47	(30,66)
	Yaoundé/Douala	82	3.4	59	(38,82)
	North	37	2.8	42	(29,58)
	South	44	3.1	43	(28,61)
Full (nonbreastfed children)*^f^*	National	50	1.9	51	(34,70)
	Yaoundé/Douala	89	1.9	61	(38,84)
	North	39	3.3	47	(34,63)
	South	44	3.0	48	(33,65)
Fortified bouillon					
SD*^d^*	National	89	0.6	0.7	(0.4,1.1)
	Yaoundé/Douala	93	1.0	0.7	(0.4,1.2)
	North	82	1.5	0.5	(0.3,1.0)
	South	93	0.7	0.7	(0.4,1.2)
Full (all children)*^e^*	National	89	1.3	1.0	(0.6,1.9)
	Yaoundé/Douala	88	2.7	1.0	(0.6,1.9)
	North	86	2.0	1.1	(0.7,2.9)
	South	91	2.1	0.9	(0.6,1.9)
Full (nonbreastfed children)*^f^*	National	91	1.1	1.1	(0.7,2.9)
	Yaoundé/Douala	90	1.8	1.0	(0.6,2.9)
	North	89	2.1	1.2	(0.8,2.9)
	South	94	1.5	1.0	(0.7,1.9)
Biofortified maize					
SD*^d^*	National	60	0.9	48	(17,101)
	Yaoundé/Douala	55	2.1	28	(12,51)
	North	58	1.7	68	(16,132)
	South	64	1.3	49	(20,100)
Full (all children)*^e^*	National	42	1.9	46	(21,84)
	Yaoundé/Douala	34	3.4	30	(13,57)
	North	45	2.9	55	(28,97)
	South	42	3.1	43	(19,81)
Full (nonbreastfed children)*^f^*	National	41	1.9	52	(26,93)
	Yaoundé/Douala	34	2.9	32	(13,61)
	North	46	3.3	62	(33,108)
	South	41	3.0	51	(25,89)
High‐dose VAS					
All*^g^*	National	90	–	–	–
	Yaoundé/Douala	90	–	–	–
	North	90	–	–	–
	South	90	–	–	–

a Because estimates from the MINIMOD‐SD tool were based on household consumption and expenditure survey data, the term *apparent* is used to emphasize that SD tool estimates were based on reported food acquisition and the assumptions that all food acquired during the recall period was consumed during the recall period without waste or food loss and that food was distributed to individual household members according to the age‐ and sex‐specific energy requirements. For the full tool, apparent consumption refers to the usual intake of food items among consumers (consumers defined in footnote *c*). Note that if an ECAM3 household had more than one member in a target group (i.e., more than one child aged 6–59 months and/or more than one WRA), we randomly selected one household member to include in the analyses of that target group. Similarly, if a household did not have a member in a specific target group, then the household was not included in analyses of that target group.

b For the SD tool estimates of reach, children of reproductive age living in households that consumed any amount in the past 7 days were counted as reached. For the full tool, reach refers to a consumption of the food by an individual in the previous 24 hours.

c Consumers were defined as those residing in a household that reported apparent consumption of the food vehicle in any quantity during the 10‐day recall period (SD estimates) or on the previous day (full tool estimates).

d Refers to estimates from the MINIMOD‐SD tool based on household consumption and expenditure survey data.

e Refers to estimates from the full MINIMOD tool based on 24HR data, estimated for children aged 6–59 months.

f Refers to estimates from the full MINIMOD tool based on 24HR data, estimated for the subset of nonbreastfed children aged 6–59 months.

g For all tools, the reach of high‐dose vitamin A supplementation was set at 90% for all regions on the basis of a 2015 external monitoring report.^45^

Abbreviations: IQR, interquartile range; SE, standard error; VAS, vitamin A supplementation.

**Figure 1:**
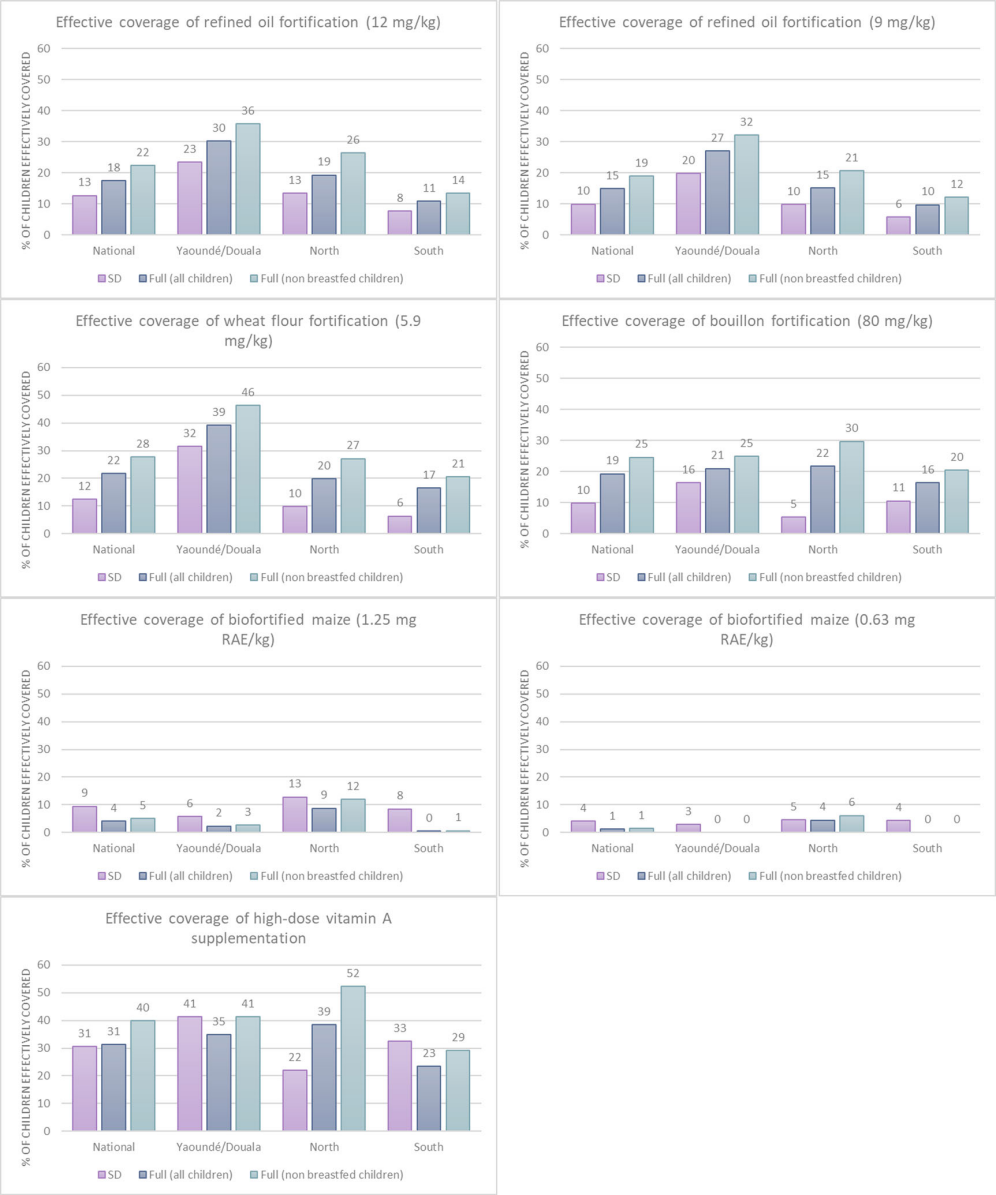
Percentage of children aged 6–59 months effectively covered by vitamin A interventions based on the MINIMOD‐SD tool using household consumption and expenditure survey data and the full MINIMOD tool using 24HR data among all children and among nonbreastfed children. Children were classified as effectively covered if they were both at risk of deficiency owing to inadequate baseline dietary intake and who also received sufficient additional intake from an intervention to be classified as having sufficient intake.

At the national level, both the SD and full tools predicted that high‐dose VAS would effectively cover the highest percentage of children (∼31% based on the SD tool and 31–40% based on the full tool) (Fig. 1 and Table S7, online only). The relative rankings of potential fortification and biofortification food vehicles based on effective coverage, however, differed somewhat across the two tools. Nationally, the SD tool predicted that VA‐fortified refined oils and fortified wheat flour would each effectively cover the highest proportion of children (∼13% and 12%, respectively), while the full tool predicted the highest effective coverage for fortified wheat flour (22–28%), followed by fortified bouillon (19–25%). Subnationally, the SD and full tool predictions of effective coverage of fortified oil and wheat flour followed the same pattern, with the percent of children effectively covered highest in Yaoundé and Douala and lowest in the South. Both tools predicted low effective coverage of biofortified maize nationally and subnationally. For VA‐fortified bouillon and high‐dose VAS, however, the SD tool subnational predictions of effective coverage were less consistent with the full tool predictions, particularly in the North.

**Figure 2:**
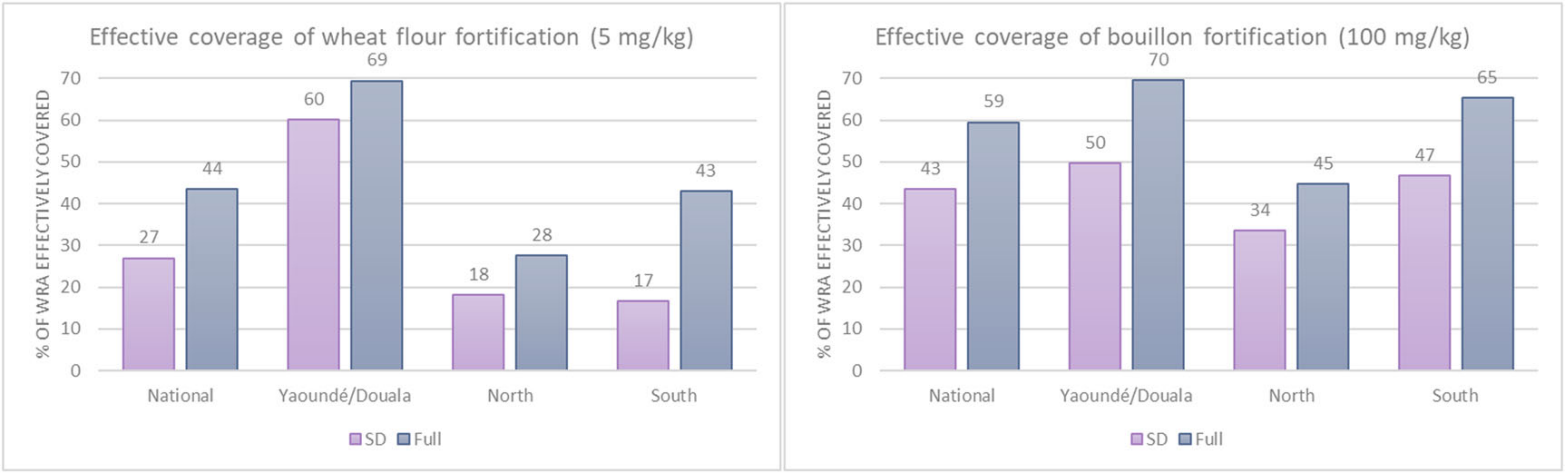
Percentage of women of reproductive age (WRA) effectively covered by folic acid interventions on the basis of the MINIMOD‐SD tool using household consumption and expenditure survey data and the full MINIMOD tool using 24HR data. WRA were classified as effectively covered if they were both at risk of deficiency because of inadequate baseline dietary intake and also received sufficient additional intake from an intervention to be classified as having sufficient intake.

Among the modeled folic acid interventions for WRA, predictions of the reach of fortified wheat flour were higher on the basis of the SD tool, while SD tool estimates of apparent daily consumption among consumers were lower than the full tool estimates (Table 3). And like for children, both tools predicted fortified bouillon would reach a high percentage of WRA (89% based on the SD tool and 93% based on the full tool), while daily apparent consumption of bouillon was slightly lower on the basis of the SD tool compared with the full tool. Both tools predicted that folic acid–fortified bouillon would effectively cover a higher percentage of WRA than wheat flour, both at the national level and in the North and South regions (Fig. 2 and Table S8, online only). In Yaoundé and Douala, predicted effective coverage of wheat flour was higher than bouillon on the basis of the SD tool (60% versus 50%), while effective coverage predictions for wheat flour (69% of WRA) and bouillon (70%) were very similar on the basis of the full tool in those cities.

**Table 3:** Reach of folic acid intervention programs and apparent*^a^* consumption of food vehicles among women of reproductive age

		Reach*b*		Apparent consumption (g/day) among consumers*c*	
Tool	Intervention	%	SE	Median	IQR
Fortified wheat flour					
SD*^d^*	National	64	0.7	26	(8,56)
	Yaoundé/Douala	96	0.5	50	(29,72)
	North	54	1.4	18	(7,53)
	South	55	1.0	13	(4,32)
Full*^e^*	National	46	1.7	73	(51,101)
	Yaoundé/Douala	78	2.4	87	(61,116)
	North	37	2.8	77	(57,101)
	South	38	2.8	61	(44,83)
Fortified bouillon					
SD*^d^*	National	89	0.5	1.6	(0.9,2.6)
	Yaoundé/Douala	90	0.9	1.8	(1.0,2.8)
	North	82	1.2	1.3	(0.7,2.4)
	South	93	0.5	1.7	(1.0,2.6)
Full*^e^*	National	93	0.9	2.0	(1.3,2.9)
	Yaoundé/Douala	92	1.6	2.1	(1.4,3.1)
	North	93	1.5	2.3	(1.5,3.2)
	South	93	1.5	1.8	(1.3,2.6)

^*a*^ Because estimates from the MINIMOD‐SD tool were based on household consumption and expenditure survey data, the term *apparent* is used to emphasize that SD tool estimates were based on reported food acquisition and the assumptions that all food acquired during the recall period was consumed during the recall period without waste or food loss and that food was distributed to individual household members according to the age‐ and sex‐specific energy requirements.

^*b*^ For the SD tool estimates of reach, women of reproductive age living in households that consumed any amount in the past 7 days were counted as reached. For the full tool, reach refers to consumption of the food by an individual in the previous 24 hours. Note that if an ECAM3 household had more than one member in a target group (i.e., more than one child aged 6–59 months and/or more than one WRA), we randomly selected one household member to include in analyses of that target group. Similarly, if a household did not have a member in a specific target group, then the household was not included in the analyses of that target group.

^*c*^ Consumers were defined as those residing in a household that reported apparent consumption of the food vehicle in any quantity during the 10‐day recall period (SD estimates) or on the previous day (full tool estimates).

^*d*^ Refers to estimates from the MINIMOD‐SD tool based on household consumption and expenditure survey data.

^*e*^ Refers to estimates from the full MINIMOD tool based on the 24HR data.

Abbreviations: IQR, interquartile range; SE, standard error.

### Micronutrient intervention costs and cost‐effectiveness

**Figure 3:**
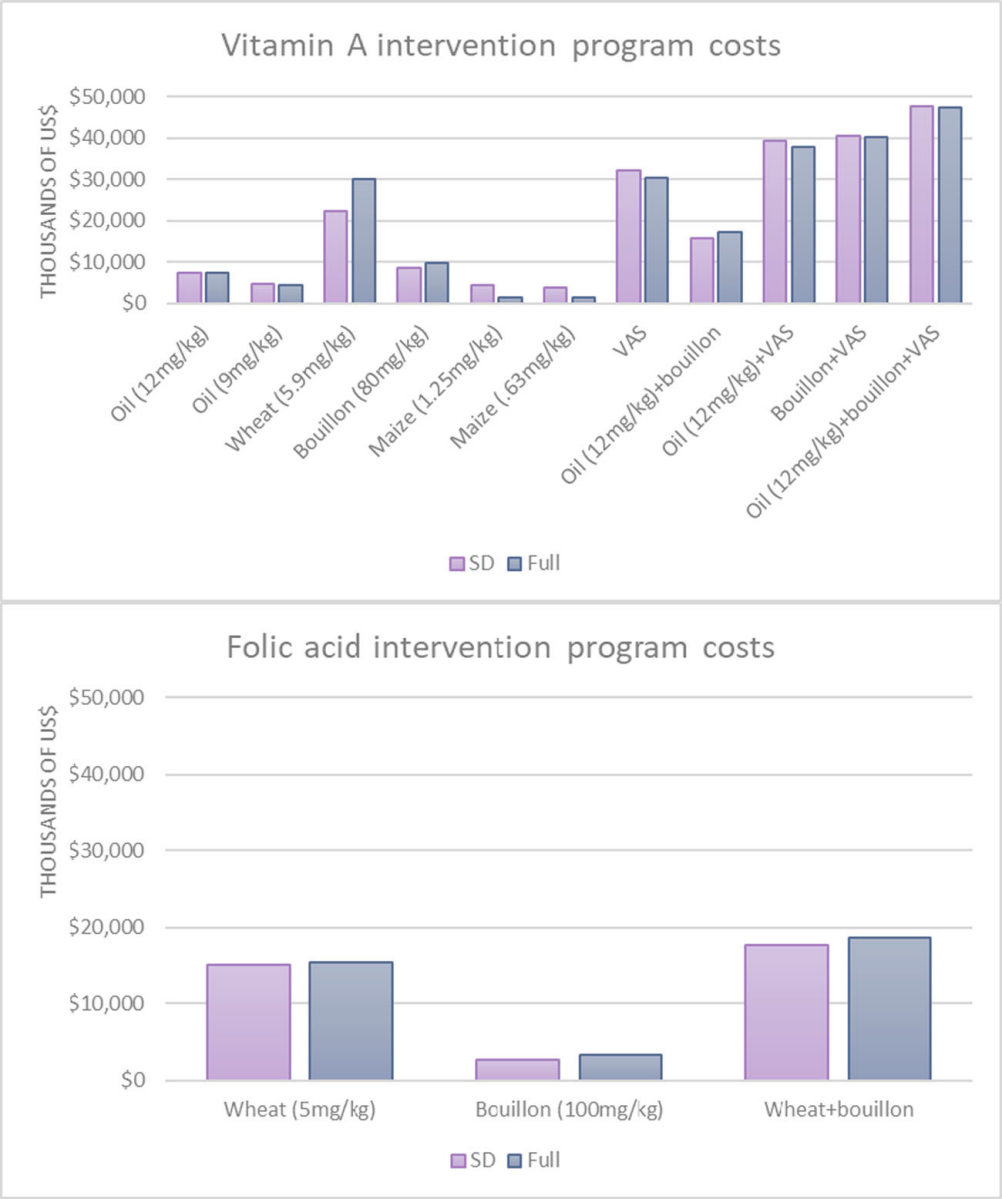
Total 10‐year (2020–2029) cost of vitamin A (top panel) and folic acid (bottom panel) intervention programs.

Overall, total 10‐year (2020–2029) cost estimates and relative rankings of interventions based on cost were very similar between the two tools (Fig. 3, top panel). The biggest differences were for VA‐fortified wheat flour (∼$22 million over 10 years based on the SD tool estimate compared with ∼$30 million based on the full tool) and biofortified maize (∼$4.5 million over 10 years based on the SD tool and ∼$1.3 million based on the full tool). We speculate about possible drivers of these differences in the Discussion section.

The estimated cost of folic acid–fortified wheat flour, bouillon cube, and the combination of the two are presented in the bottom panel of Figure 3. The SD tool estimates of the 10‐year costs of delivering folic acid via these food fortification vehicles were very similar to the full tool estimates (Fig. 3, bottom panel).

**Figure 4:**
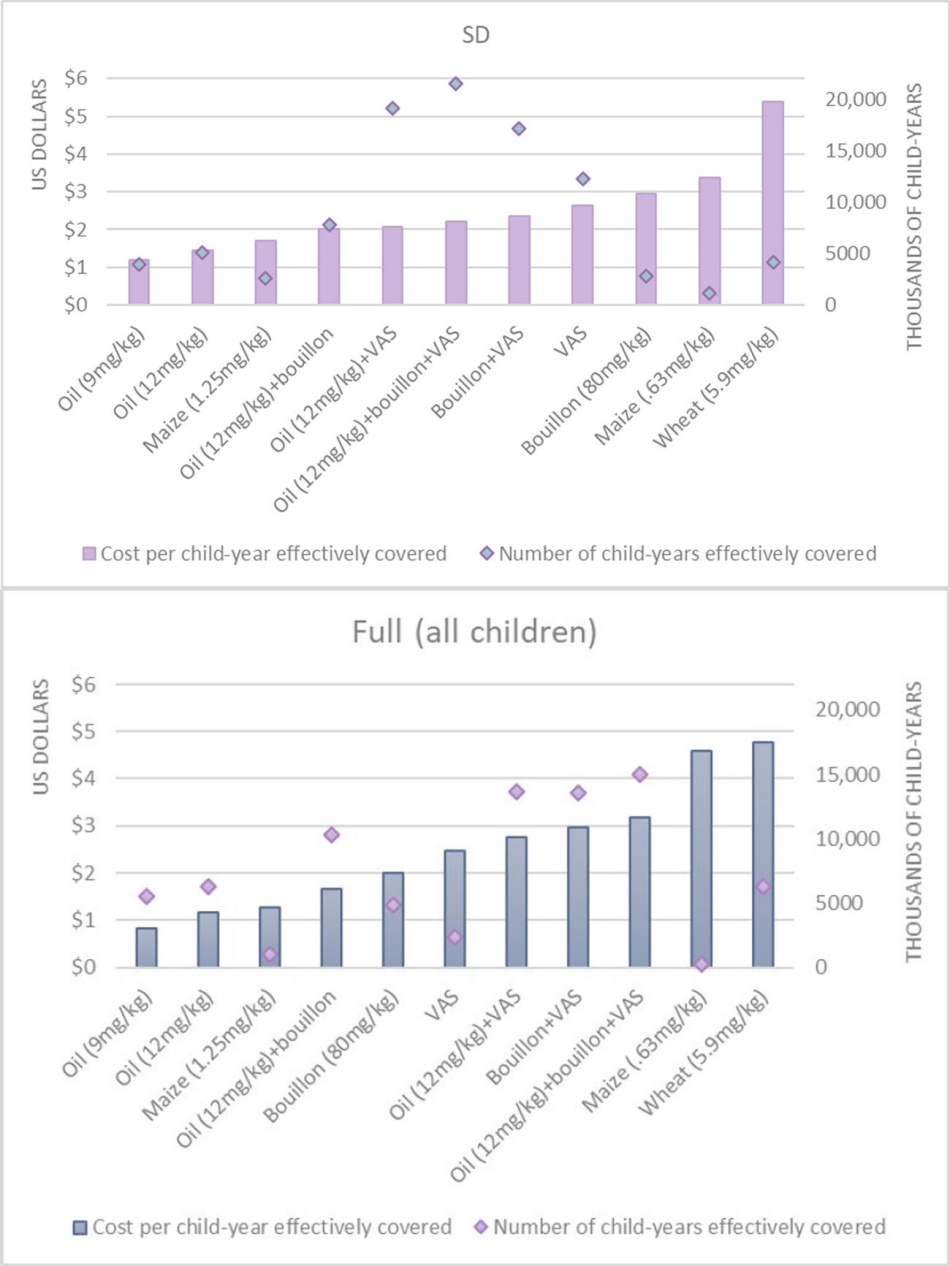
Estimated number of children aged 6–59 months effectively covered by vitamin A intervention programs, and select combinations of programs, and estimated undiscounted cost per child effectively covered (measured in child‐years over 10 years) on the basis of the MINIMOD‐SD tool (top panel) using household consumption and expenditure survey data and other secondary data, and the full MINIMOD tool (bottom panel) using 24HR data and other primary data sources.

At the national level based on SD and full tool estimates of effective coverage and cost, VA‐fortified edible oil was predicted to be the most cost‐effective individual intervention, followed by biofortified maize and then the combination of fortified oil and bouillon (Fig. 4 ; see Table S9 for subnational results, online only). Beyond these most cost‐effective interventions, the two tools diverged in the rankings of bouillon, VAS, and other combinations. Finally, both tools predicted that VA‐fortified wheat flour was substantially less cost‐effective than the other individual or combined interventions.

**Figure 5:**
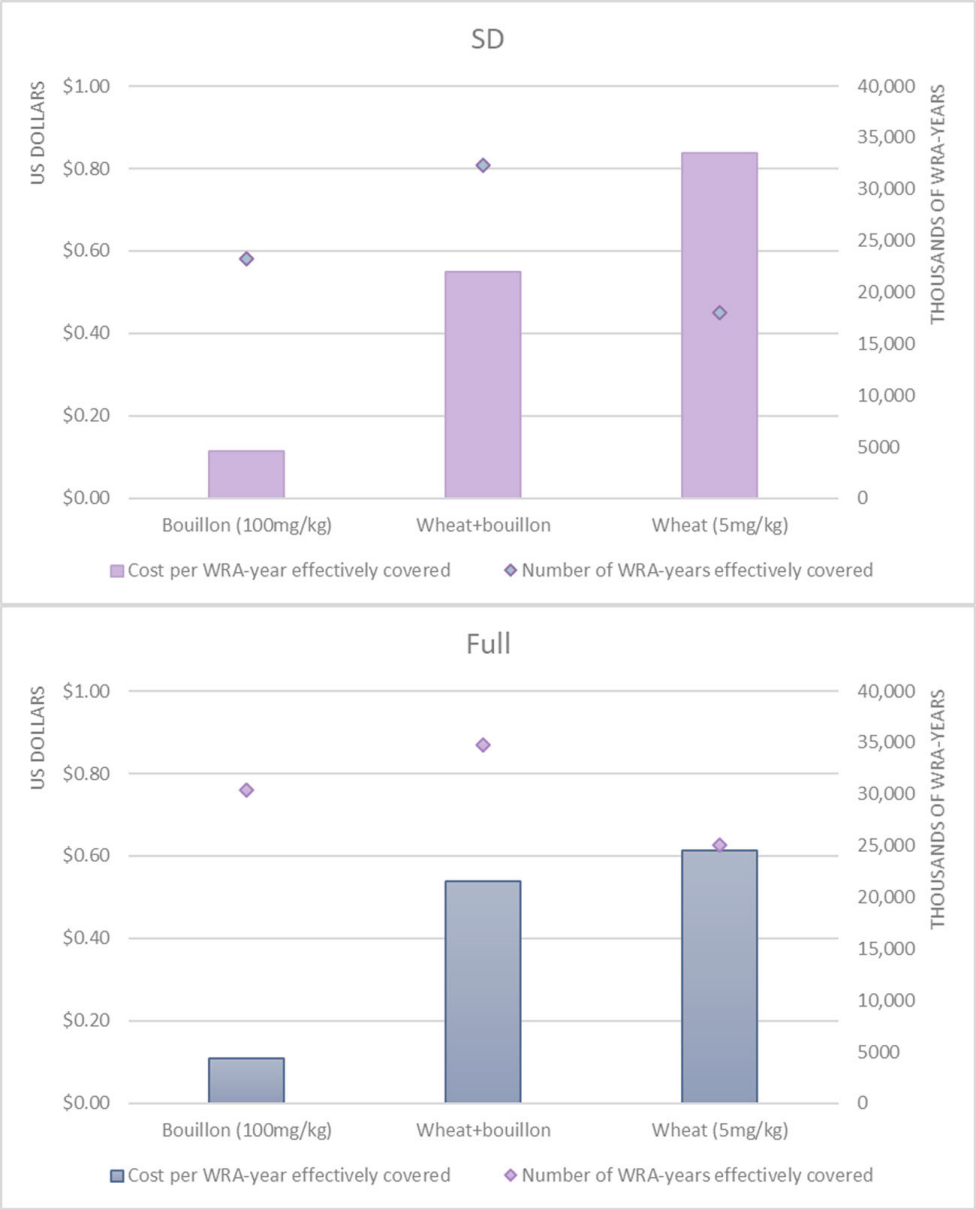
The estimated number of women of reproductive (WRA) age effectively covered by folic acid intervention programs, and combinations of programs, and estimated undiscounted cost per WRA effectively covered (measured in WRA‐years over 10 years) on the basis of the MINIMOD‐SD tool (top panel) using household consumption and expenditure survey data and other secondary data, and the full MINIMOD tool (bottom panel) using 24HR data and other primary data sources.

The total number of WRA effectively covered by folic acid fortification alongside the estimated cost per WRA effectively covered are presented in Figure 5, again ordered by cost‐effectiveness. At the national level, both tools predict that folic acid–fortified bouillon would be substantially more cost‐effective than fortified wheat flour (and the combination of bouillon and wheat flour) at the national level (see Table S10 for subnational results, online only).

## Discussion

Given the dearth of individual‐level, nationally representative dietary intake data in LMICs, we developed the MINIMOD‐SD tool to provide policymakers in LMICs with a systematic framework for estimating the need for, and for predicting the impacts, cost, and cost‐effectiveness of alternative micronutrient intervention programs using available secondary data. To identify the types of policy questions that analyses of secondary data using the SD tool methodology may or may not be suited to inform, in the context of Cameroon, we compared the SD tool estimates with those generated via the full MINIMOD tool, which used the 24HR and primary cost data. Before summarizing the results of this comparative analysis and discussing the implications for using the SD tool methodology, we first outline the primary limitations imposed by using secondary data, as these provide context for differences observed between the two tools.

First, as previously noted, food data from HCESs often reflect food acquisition or expenditure rather than food consumption, which may vary substantially, particularly for food items that may be purchased in bulk (e.g., condiments) and have a long shelf‐life. Also, quantities of food acquired or purchased are often reported in nonstandard units, posing a challenge if conversion factors are unavailable. In this analysis, to convert nonstandard unit quantities to grams, we used imputed median price‐per‐gram estimates on the basis of the total amount paid for the acquisition of food reported in standard units in the ECAM3 data. If the prices paid by households that chose to report quantities in standard units were systematically different than prices faced by households that primarily chose nonstandard units, the imputed price‐per‐gram estimates and, therefore, estimates of apparent food consumption and nutrient intake may be biased.^46^

Another limitation is that HCES data are collected at the household level, so estimates of food consumption and nutrient intake at the individual level depend on assumptions about the intrahousehold distribution of food. Also, FAFH are oftentimes inadequately captured, or not captured at all, in the HCES data. In our HCES analysis, we were unable to account for FAFH, although FAHF captured in the survey was only ∼3% of reported total daily household food expenditures nationally, ranging from ∼2% in the North to ∼4% in the cities of Yaoundé and Douala. Another limitation is that HCES data do not typically distinguish breastfed from nonbreastfed children, so energy and nutrient intake from breastmilk are either unaccounted for or must be estimated. Finally, we note that the risk of dietary inadequacy, even when measured at the individual level, may not accurately reflect the prevalence of deficiency as determined by micronutrient biomarkers (e.g., owing to the misestimation of micronutrient absorption or the effect of infections on micronutrient metabolism). Examination of data on micronutrient status and related information, such as the prevalence of stunting or anemia, may facilitate the interpretation of estimates of dietary intake or household consumption data for nutrition program decision making.

In the context of these limitations, we return to the set of questions we set out to answer and reflect on the extent to which the SD tool estimates are an adequate alternative to the full tool estimates when 24HR data and/or detailed intervention cost information are unavailable. The first question was whether the nutrition need estimates derived from the SD tool suggested the same problems as the full tool and whether the spatial patterns of inadequate micronutrient intake were similar between the two tools. For VA, the SD tool estimates of apparent dietary intake were lower than the full tool estimates, leading to an overestimate of the prevalence of inadequate VA intake among children relative to the full tool (75% compared with 49% based on the full tool). Although the SD tool estimates of inadequate VA intake overstated the problem relative to the full tool in each of the three macroregions, the tools were consistent in identifying the North as the macroregion with the highest proportion of children at risk of VA deficiency because of inadequate intake and the South as the region with the lowest risk. When we compared the SD tool estimates with the full tool estimates on the basis of the subset of nonbreastfed children, the magnitude of the difference between the tools decreased, suggesting that not accounting for VA intake from breastmilk might explain some of the difference in intake and inadequate intake between the two tools but that other foods “missed” in the HCES data or misallocated within the household based on the AME method (e.g., complementary foods) may also be responsible for some of the difference. For folate, both the estimates of apparent intake and the prevalence of inadequate intake among WRA were very similar between the two tools, and the subnational pattern of inadequate intake based on the SD tool was consistent with the full tool. Taken together, these results suggest that policymakers could reliably use the SD tool to inform policy decisions about the need for interventions to address inadequate VA or folate intake and where the needs are greatest, with the caveat that the prevalence of inadequacy may be overestimated, particularly for young children.

Second, we examined the similarity of the national and subnational rankings of micronutrient interventions on the basis of the nutrition benefit estimates derived from each tool. To answer this question, we compared estimates of intervention reach, consumption of fortifiable/biofortifiable foods, and intervention effective coverage. For interventions to deliver VA to children as well as interventions to deliver folic acid to WRA, we found that the SD tool estimates of the reach of fortifiable/biofortifiable foods were generally higher than the full tool estimates, while estimates of apparent intake of these foods among consumers were lower, and sometimes substantially so, on the basis of the SD tool compared with the full tool. These differences may be partly attributed to the underlying data collection methods. Because the HCES survey collected data on food acquisitions over a 10‐day period and represented food acquisitions at the household level, the likelihood that a fortifiable/biofortifiable food was reported as acquired by the household in any amount over those 10 days may be higher than the probability that food was consumed by a child/woman in the 24 h before the recall. And, because average daily consumption among consumers in the HCES data was calculated as the average over the 10 days, this may have contributed to lower estimates of average apparent consumption among consumers.

With reference to effective coverage of VA intervention programs, we found that both tools identified VAS as the intervention with the highest effective coverage and biofortified maize at 0.63 mg RAE/kg as the intervention with the lowest effective coverage. And although there was some variability in the relative rankings of the other fortification and biofortification interventions to deliver VA to children, at the national level, the range of predictions of effective coverage were fairly tight (from 9% to 13% for the SD tool and from 5% to 22% for the full tool), suggesting that both tools did not predict much variability in the effective coverage of these interventions. For folic acid interventions, the two tools were consistent in identifying fortified bouillon as effectively covering a higher percentage of WRA than fortified wheat flour at the national level, although for bouillon and wheat flour, the SD tool estimates of effective coverage were consistently lower than the full tool. Given these results, the SD tool estimates may reliably help policymakers identify interventions that are likely to be most and least effective for specific target populations, but quantitative estimates of effectiveness and subnational variation in effectiveness may require individual‐level dietary data.

Finally, we asked how similar the estimates of intervention program cost‐effectiveness were across the two tools. Among the modeled VA interventions, there was a large discrepancy between the two tools in the estimated cost of the VA‐biofortified maize program (although both tools still identified biofortified maize at 100% replacement as among the most cost‐effective interventions). Specifically, the 10‐year cost of the biofortified maize intervention was estimated to be more than three times more expensive on the basis of the SD tool compared with the full tool. Biofortified maize is not currently implemented in Cameroon, so both sets of cost estimates were hypothetical in nature. The SD tool cost estimates were based on estimates from the literature^22^, ^47^ that were adapted to the Cameroonian context to the extent possible, while the full tool estimates were based on input from experts in Cameroon.^8^ For estimating intervention program costs, while the SD tool estimates were generally in line with the full tool cost estimates, supplemental primary data collection would be beneficial. In particular, supplementing and sense‐checking the SD tool cost model structures, assumptions, and unit cost estimates via interviews and/or workshops with in‐country experts could be a low‐cost way to improve the validity of those estimates.

Estimates of the cost‐effectiveness of other VA interventions and select combinations of interventions led to similar national rankings of the interventions, with some small differences but overall agreement in cost‐effective versus less cost‐effective interventions. Overall, among individual and combined interventions, VA‐fortified refined oil was identified as a very cost‐effective intervention to effectively cover children aged 6–59 months, both nationally and subnationally, at both current and target fortification levels. Both tools also identified biofortified maize and the combination of VA‐fortified oil and bouillon as the next most cost‐effective interventions. VA‐fortified wheat flour was clearly the least cost‐effective according to both tools, though it is important to note that the cost of the wheat flour fortification program reflected the cost of fortification with VA plus the four other micronutrients that are included in Cameroon's wheat flour standard (iron, folic acid, zinc, and vitamin B_12_). For folate, both tools clearly identified folic acid–fortified bouillon as the most cost‐effective intervention to effectively cover WRA, followed by the combination of fortified bouillon and wheat flour. The SD tool rankings of interventions based on predicted cost‐effectiveness may help policymakers, operating in resource‐constrained environments, determine where resources would likely be most efficiently allocated (i.e., how to effectively cover the greatest number of children or WRA per dollar spent).

For several reasons, the SD and full tool estimates are not directly comparable. First, each tool relied on a different sample of households for the underlying apparent food consumption/dietary recall data. This is in contrast with several other efforts to compare the use of HCES to 24‐h data that have based their comparisons on the same samples.^48^, ^49^, ^50^, ^51^, ^52^ Also, the HCES data were collected 2 years before the 24‐h data, although both surveys were conducted during the same seasons. With these differences in mind, taken together, the comparative results suggest that overall, the SD tool estimates based on HCES data are better proxies for intake among WRA compared with children, as the degree of discrepancy between the intake and prevalence of inadequate intake estimates was generally smaller for WRA than children. This is consistent with other studies that have also found less agreement between the HCES and 24HR estimates among children compared with other population groups.^51^, ^53^ For both children and WRA, we found that the SD tool consistently underestimated micronutrient intake and overestimated the prevalence of inadequate intake. However, qualitatively for both WRA and children, there was general agreement between the two tools in where in the country the risks of deficiency were greatest and the most effective and cost‐effective interventions to reduce the prevalence of inadequate intakes.

Overall, relying on secondary data and the SD tool methodology may help policymakers to assess the extent to which diets are providing adequate micronutrients and characterize spatial patterns of inadequate micronutrient intakes, which may help them decide if and where interventions may be needed. The SD tool estimates of the relative reach and consumption of fortifiable and biofortifiable foods, combined with predictions of the most cost‐effective interventions or sets of interventions, can help policymakers to determine which interventions are likely to efficiently provide effective coverage for target populations and facilitate necessary discussions of cost sharing among stakeholder groups. However, accurately quantifying the extent of inadequate intakes for individual household members and intervention effectiveness and identifying relatively small differences in cost‐effectiveness across interventions nationally and, especially subnationally, will likely require individual‐level dietary recall data. As these conclusions are based on the evaluation of the results for two micronutrients assessed for a single country, further research is necessary to understand the generalizability of these results to other micronutrients and contexts. Additionally, if policymakers wanted to compare these results to the cost‐effectiveness of other health interventions or to a standard threshold for cost‐effectiveness, further work would be required to translate these estimates into common health metrics, such as disability‐adjusted life years averted or lives saved. Our future work aims to assess the performance of the SD tool for a wider range of micronutrients (including iron, zinc, and vitamin B_12_), for estimating functional outcomes, including cases of anemia averted and lives saved, and for identifying economically optimal sets of interventions over space and time using the MINIMOD economic optimization framework.

## Author contributions

K.P.A., S.A.V., and R.E.S. designed the study and developed the methods. K.P.A., H.L., and J.K. analyzed and modeled the data and accept responsibility for the integrity of the data analyzed. K.P.A. wrote the first draft of the manuscript. All authors contributed to the data interpretation and revisions of the manuscript and read and approved the final manuscript.

## Competing interests

The authors declare no competing interests.

## Supporting information


**Figure S1**. The MINIMOD‐SD tool process.
**Figure S2**. Summary HCES data preparation and analysis steps for use in the MINIMOD‐SD tool.
**Table S1**. Reference values by target group and micronutrient.
**Table S2**. Modeled micronutrient intervention program assumptions.
**Table S3**. Default fortification program cost activities.
**Table S4**. Default biofortification program cost activities.
**Table S5**. Potential data sources to inform industry and program information and unit costs for a cost model.
**Table S6**. Estimated apparent energy intakes.
**Table S7**. Predicted effective coverage of individual and select combinations of vitamin A interventions: Children aged 6–59 months.
**Table S8**. Predicted effective coverage of individual and select combinations of folic acid interventions: WRA.
**Table S9**. National and subnational nutrition benefits, costs, and cost‐effectiveness of alternative vitamin A intervention programs over 10 years (2020–2029).
**Table S10**. National and subnational nutrition impacts, costs, and cost‐effectiveness of alternative folic acid intervention programs over 10 years (2020–2029)
