# Supplementary material for: Comparing estimated cost‐effectiveness of micronutrient intervention programs using primary and secondary data: evidence from Cameroon
Source: Ann N Y Acad Sci. 2021 Dec 9;1510(1):100–20. doi: 10.1111/nyas.14726 (PMC9299899; doi:10.1111/nyas.14726)
Supplement: Supplementary file 1 — Figure S1. The MINIMOD‐SD tool process. Figure S2. Summary HCES data preparation and analysis steps for use in the MINIMOD‐SD tool. Table S1. Reference values by target group and micronutrient. Table S2. Modeled micronutrient intervention program assumptions. Table S3. Default fortification program cost activities. Table S4. Default biofortification program cost activities. Table S5. Potential data sources to inform industry and program information and unit costs for a cost model. Table S6. Estimated apparent energy intakes. Table S7. Predicted effective coverage of individual and select combinations of vitamin A interventions: Children aged 6–59 months. Table S8. Predicted effective coverage of individual and select combinations of folic acid interventions: WRA. Table S9. National and subnational nutrition benefits, costs, and cost‐effectiveness of alternative vitamin A intervention programs over 10 years (2020–2029). Table S10. National and subnational nutrition impacts, costs, and cost‐effectiveness of alternative folic acid intervention programs over 10 years (2020–2029) [file NYAS-1510-100-s001.docx]

**Online Supporting Material**

Comparing estimated cost-effectiveness of micronutrient intervention programs using primary and secondary data: Evidence from Cameroon

**Supplementary Methods and Tables**

*MINIMOD-SD tool overview*

Figure S1 below depicts the MINIMO-SD tool process and underlying models. The process starts with the identification of demographic and nutrition secondary data sources as well as secondary cost data sources. Examples of potential secondary data sources are listed in the blue boxes. Once secondary data sources have been identified, the data generally need to be prepared and processed for use in the models. This step generally requires some level of technical expertise and considerable time and effort. On the nutrition side, the data processing leads to population projections (at the national and sub-national levels, for each target population of interest, for each year of the planning time horizon) as well as estimates of food and dietary nutrient intake, the prevalence of inadequate intake, and the reach of alternative intervention programs. On the cost side (further detailed in the section below), secondary data are processed to arrive at input (or “ingredient”) cost estimates for all activities that are assumed to be undertaken for each potential intervention program.

Estimates based on the processed secondary data are then entered into an Excel-based template that houses both the nutrition needs and benefits model and the cost model. The nutrition needs and benefits model estimates nutrition needs (based primarily on the estimated prevalence of inadequate dietary micronutrient intake) and simulates the potential benefits, at the national and sub-national level and for each year of the 10-year time horizon, of each potential intervention (and all combinations) in terms of the measures of nutrition benefits (the general steps in using HCES data to estimate nutrition needs and model benefits are listed in Figure S2 below).

Total undiscounted and discounted nutrition benefits are presented for each intervention and combinations of interventions. On the cost side, estimated input costs (e.g., the cost per kg of micronutrient premix for fortification) for each intervention are entered into costing templates that are organized by activity (e.g., training, internal and external quality control, industrial inspections), by type of cost (start-up or operating), by year, and by cost bearer (e.g., industry or the government). The sub-total cost of all activities for each specific intervention are summed, by year and over the 10-year time horizon, to arrive at total cost estimates. Undiscounted and discounted total cost estimates for each intervention are presented in base year US dollars.

Within the Excel template, the total 10-year nutrition benefits and intervention cost estimates are then joined to estimate and compare the cost-effectiveness of each intervention and combinations of interventions at the national and sub-national levels.

**Demographic and nutrition data preparation/processing (Stata, Excel)**

- Population projections
- Apparent food consumption
- Estimates of dietary micronutrient intake
- Prevalence of inadequate and high intake

**Identification of secondary demographic and nutrition data sources**

- Household consumption and expenditure surveys
- Food composition tables
- National census
- United Nations population projections
- Demographic and Health Survey
- Multiple Indicator Cluster Surveys
- Lives Saved Tool (LiST)
- Activity-specific input cost estimates

**Cost data preparation/processing (Excel)**

**Identification of secondary cost data sources**

- WHO-CHOICE
- OneHealth Tool
- UNICEF supply catalog
- Food Fortification Initiative
- Global Fortification Data Exchange
- FAO food balance sheets
- World Bank Development Indicators
- Trade association reports
- Literature

*1*

Figure S1. The MINIMOD-SD tool process

- Predicts nutrition benefits (reach, effective coverage, cases of anemia averted) and intake above the UL for all combinations of candidate interventions
- Spatially and temporally explicit

**Cost-effectiveness (Excel)**

- Summary estimates of annual and 10-year total benefits and costs
- Cost per person in the target population effectively covered over 10-year time horizon

**Intervention program cost model (Excel)**

- Estimates government and industry start-up and operating costs for all combinations of candidate interventions
- Spatially and temporally explicit

**Nutrition needs and benefits model (Excel)**

**Steps in HCES data preparation and analysis**

1. Identify data sources nutrient values and match foods in HCES food list to FCT entries
2. Select dietary reference values for each micronutrient of interest
3. Determine equivalence factors for fortifiable foods contained in processed foods
4. Calculate adult male equivalent (AME) factors
5. Standardize food consumption/expenditures to a common unit of measure (e.g., grams)
6. Calculate daily apparent household consumption of each food
7. Estimate total daily household apparent micronutrient intake
8. Use AME factors to estimate apparent individual food consumption and micronutrient intake
9. Compare apparent intake to reference values to estimate the prevalence of inadequate and high apparent intake from diets
10. Estimate consumption of (bio)fortifiable food vehicles of interest and receipt of supplements
11. Model the impact of (bio)fortification on the prevalence of inadequate and high apparent intake by adding apparent (bio)fortifiable food consumption multiplied by assumed level of (bio)fortification to dietary intake and recalculating the prevalence of inadequate and high apparent intake
12. Model the impact of supplementation on the prevalence of inadequate and high apparent intake by adding the daily equivalent intake via supplemetation to dietary intake and reclaculating the prevalence of inadequate and high apparent intake

Figure S2. Summary HCES data preparation and analysis steps for use in the MINIMOD-SD tool

*ECAM3 sampling strategy and data collection*

Data collection for ECAM3 (Troisième Enquête Camerounaise auprès des Ménages) was conducted by the Cameroon National Institute of Statistics from September through December of 2007. The sampling design was a two-stage stratified random sample with 32 strata; each of the 10 regions was divided into three strata (urban, semi-urban, and rural) and the two main cities, Yaoundé and Douala, were treated as separate stratum (Republique du Cameroun Institut National de la Statistique, 2007). Data on household food expenditures and acquisitions were collected twice from rural households. During the first visit, households were asked to recall all food acquired, via purchase, home production, as a payment, or as gift, over the period of the past seven days. Four days later, rural households were visited again and asked about daily food acquisitions on each of the previous three days. Urban and semi-urban households were given a notebook to record their food acquisitions, and these households were visited approximately every three days for a period of 18 days to collect data from their notebooks. Because rural households had a maximum of ten days of recall while urban and semi-urban households had 15 days of recall, we used the first ten days of recall from all households to make the number of days of recall included in the analysis equal across all households.

Table S1. Reference values by target group and micronutrient

| **Micronutrient** | **Target group** | **Estimated average requirement (EAR)** | **Tolerable upper intake level (UL)** |
| --- | --- | --- | --- |
| Vitamin A^1^ | Children, 1 - 3 years | 210 µg RAE/d | 600 µg/d preformed retinol |
|  | Children, 4-5 years | 275 µg RAE/d | 900 µg/d preformed retinol |
| Folate^2^ | Pregnant WRA, 15-18 years | 520 µg DFE/d | 800 µg/d folic acid |
|  | Pregnant WRA, 19-49 years | 520 µg DFE/d | 1000 µg/d folic acid |
|  | Non-pregnant WRA, 15-18 years | 330 µg DFE/d | 800 µg/d folic acid |
|  | Non-pregnant WAR, 19-49 | 320 µg DFE/d | 1000 µg/d folic acid |

RAE, retinol activity equivalents; DFE, dietary folate equivalents; WRA, women of reproductive age.

^1^The estimated average requirement (EAR) and tolerable upper level (UL) values for vitamin A were from the US Institute of Medicine (IOM) (Institute of Medicine, 2001). The EAR for vitamin A intake is not defined for children 6-12 months old. In this analysis, we used the EAR for children 1-3 years of age (210 µg RAE/day) for children 6-12 months.

^2^The EAR values for folate were from the US Institute of Medicine (Institute of Medicine, 1998). The tolerable upper intake level (UL) for folic acid was defined based on folic acid intake from fortified foods and supplements only, as there is no UL for folate from naturally occurring food sources (Institute of Medicine, 1998).

Table S2. Modeled micronutrient intervention program assumptions

| Micronutrient | Intervention program | Modeling assumptions |
| --- | --- | --- |
| Vitamin A | Refined oil fortification | Scenario 1: Fortified at 12 mg/kg^1^ |
|  |  | Scenario 2: Fortified at 9 mg/kg^1^ |
|  | Wheat flour fortification | Fortified at 5.9 mg/kg |
|  | Bouillon fortification | Fortified at 80 mg/kg |
|  | Biofortified maize | Scenario 1: Biofortified at 1.25 mg RAE/kg^2^ |
|  |  | Scenario 2: Biofortified at 0.63 mg RAE/kg^2^ |
|  | High-dose vitamin A supplementation | Children 6-12 mo: 30 mg once per year |
|  |  | Children 12-59 mo: 60 mg every 6 mo |
| Folic acid | Wheat flour fortification | Fortified at 5 mg/kg |
|  | Bouillon fortification | Fortified at 100 mg/kg |

RAE, retinol activity equivalents; WRA, women of reproductive age.

^1^The oil fortification standard in Cameroon is 12 mg/kg (Global Fortification Data Exchange, 2020), while the average current level of fortification is 9 mg/kg (Mark, Assiene, Luo et al., 2019).

^2^ Under scenario 1, the modeling assumption was that all maize production in Cameroon was replaced with a biofortified variety that provided 1.25 µg RAE of vitamin A per gram of maize. Under scenario 2, the modeling assumption was that half of the maize in Cameroon was replace with the biofortified variety that provided 1.25 µg RAE of vitamin A per gram of maize, or, equivalently, the biofortified variety was assumed to provide an average of 0.63 µg RAE of vitamin A per gram of maize.

*MINIMOD-SD tool micronutrient intervention program cost models*

For each potential intervention, 10-year cost models are developed to estimate start-up and recurring costs faced by industry (if relevant) and the government associated with planning, implementing, and operating the intervention. For fortification and biofortification interventions, cost models are developed and estimated using an activity-based approach. That is, for each intervention program, the cost model is structured based on the set of activities required to plan, execute, and manage the intervention. Each activity is then populated with a series of inputs (or “ingredients”) that go into performing each activity and the estimated cost associated with each input. For example, for fortification interventions, internal quality assurance/quality control activities faced by industry (mills, factories, refineries) might include supplies for chemical analysis as well as labor (e.g., lab technicians). For supplementation interventions, a modified activity-based approach is used (described in detail below). In short, this modified approach follows the Lives Saved Tool (LiST) costing methodology (Bollinger, Sanders, Winfrey et al., 2017) and involves estimating unit costs, by visit/contact, for the inputs associated with undertaking activities directly associated with the delivery of supplements, while additional facility-level direct and indirect costs are estimated as a proportion of the cost associated with each out-patient visit (based on country-specific estimates made by the World Health Organization and available at WHO-CHOICE website (<http://www.who.int/choice/cost-effectiveness/en/>), while program-level (i.e., above facility-level) costs, including supervision, transportation, communications/outreach, overall program management, etc., are each estimated as a percentage of total cost per visit/contact.

To the extent possible, cost estimates for each input (or unit costs) are derived from secondary data sources and from published and grey literature (where, for example, unit costs estimated from a similar intervention in a similar setting are presented and can be adapted for the SD tool focus country). In most cases, it is advisable to sense-check or verify unit cost estimates sourced from secondary data and the literature with input from international and local experts and/or existing program budgets where they exist. In some cases, it may be necessary to supplement secondary cost data with primary data collection in order to get realistic, up-to-date estimates of some intervention activities and/or unit costs.

To date, the SD tool has been used to estimate the costs of fortification, biofortification, and supplementation interventions. Each of these categories of cost models are described, in turn, below.

*(a) Fortification cost models*

Estimating the costs of fortifying foods and condiments with single or multiple micronutrients first involves characterizing the industry. This includes information about the number of industrial-scale processing facilities (mills, refineries, factories, etc.) where fortification occurs, the number of days of operation per year, and the average wage of facility personnel who would be involved in the fortification process or internal quality control and quality assurance activities. This information feeds into calculations of specific industry startup-up and operating costs. At the government level, fortification cost estimates rely on characteristics of the existing or hypothetical monitoring and evaluation (M&E) plan undertaken by the government to assess whether the fortification program is meeting its objectives and to evaluate whether changes to the fortification program are necessary (2006). If some of the fortified food/condiment are imported, the characteristics of the import industry (number of ports of entry, etc.) are also relevant, and costs of monitoring at boarders is also accounted for.

Table S3 shows the activities that comprise the SD tool default industry and government fortification cost models. Among industry start-up costs, which are generally assumed to take place in the first few years of the 10-year time horizon unless fortification of the specific food vehicle is already in place, equipment needs are food-vehicle-specific, but potentially include the purchase of equipment to carry out the fortification process (e.g., mixing tanks, pumping systems, feeder/dosifier, blender) as well as equipment and supplies (e.g., iCheck test kits and reagents) to carry out internal quality control activities. We also assume that industry will be required to pay for the redesign of the label(s) of their fortified products to indicate that the product is fortified. It is also assumed that production facilities will conduct training and sensitization for fortification and quality assurance personnel during the start-up period.

Table S3. Default fortification program cost activities

| **Type of cost** | **Industry** |  | **Government** |
| --- | --- | --- | --- |
| Start-up | Equipment |  | Planning |
|  | Label redesign |  | Equipment |
|  | Training |  | Social marketing and advocacy |
|  |  |  | Training |
| Recurring | Premix acquisition |  | Factory/mills inspections and monitoring |
|  | Fortification |  | Subsidization of imported fortified food |
|  | Internal QA/QC |  | Import monitoring |
|  | External QA/QC |  | Commercial monitoring/market surveys |
|  | Training/retraining |  | Household monitoring |
|  | Management, overhead, and admin. |  | Social marketing |
|  |  |  | Nutrition surveillance |
|  |  |  | Training/retraining |

QA/QC: quality assurance/quality control;

Government start-up costs are assumed to involve several planning activities spread over the start-up period, including conducting a baseline survey and market assessment, formulation of fortification standards/norms, and the development of an M&E plan. The government is also assumed to face start-up equipment costs related to monitoring activities (e.g., iCheck test kits) and to conduct a social marketing/advocacy campaign as well as training for food control agency/program monitors, lab technicians, and supervisors.

The recurring costs faced by industry are the cost of acquiring the micronutrient premix (or acquiring/producing fortified rice kernels in the case of fortified rice), which are calculated based on the assumed annual quantity of domestically fortified product multiplied by the premix cost (including shipping, storage, etc.) per metric ton of food vehicle. It is also assumed that production facilities hire additional labor to conduct fortification-related production activities as well as pay for annual maintenance of their fortification equipment. Industry quality control/quality assurance activities are also included in the cost models, where we assume that production facilities conduct several in-house tests of the micronutrient content of their food product, which requires chemical reagents and the time of in-house lab technicians. We also assume that samples of fortified food/condiments are regularly sent for external quantitative testing.

Recurring government costs are primarily related to M&E activities. For domestically fortified foods/condiments, we assume the government conducts regular site-visits to the local production facilities for monitoring and to collect samples for testing. If some of the product is assumed to be imported, the government also faces import monitoring costs at each port of entry, including the cost of conducting regular inspections at each port of entry and chemical analysis of imported samples. Government recurring costs may also include market surveys to collect and analyze fortified food samples sold in markets as well as household monitoring. We also assume that the government will invest in social marketing/advocacy activities for several years after start-up and conduct a follow-up national nutrition survey approximately every five years after start-up.

The final cost component is the cost of imported fortified food/condiments. The default cost models estimate the cost of imported fortified product as the total annual cost of premix needed to fortify the volume of imported product.

Some components of the costs of domestic and foreign (i.e., imported) fortification depend on the assumed proportion of potentially fortifiable food/condiment that is actually fortified. Assumptions about the scale of fortification are built directly into the cost models at an annual time-step such that scale-up can occur over multiple years and/or fortification programs operating “below capacity” can be reflected in the cost estimates. Undiscounted and discounted total fortification costs are estimated at the national level and then allocated to each subnational unit according consumption-based weights. That is, the share of the total cost of the fortification program allocated to each subnational unit is calculated as the total cost weighted by the subnational unit’s share of total consumption of the potentially fortifiable food or condiment.

Cost models are built separately for each potential food vehicle. To estimate the cost of two or more fortification vehicles together (if, for example, wheat flour and bouillon cube are both fortified with iron), costs are assumed to be additive except for government startup costs and the cost of government nutrition surveillance, which are both added to the total cost only once.

*(b) Biofortification cost models*

The SD tool biofortification cost models being with information that characterizes the production and supply of the biofortifiable crop. This includes information about the number of regions and/or districts where the crop is grown (which has implications for the assumed number of field schools and agricultural extension workers), the availability of the crop in the food supply, and the average wage of extension workers and field school operators.

Table S4 shows the activities that comprise the SD tool default biofortification cost models, which are all assumed to be borne by the government. Start-up activities, which are generally assumed to take at least several years, include planning (conducting a baseline nutrition survey, policy engagement, development of an M&E plan, etc.) as well as planting material (seeds, vine cuttings, etc.) multiplication and dissemination. Note that the original R&D costs associated with the development and release of appropriate varieties of the crop are assumed to have been previously incurred and are thus zero in the cost models. Sensitization, marketing, and education/advocacy campaigns are also assumed to happen during start-up, along with the establishment of field schools, the training of ag extension workers and field school operators and conducting demonstration days at the field schools during the planting and harvesting seasons, which are all assumed to occur during the final few years of start-up.

Table S4. Default biofortification program cost activities

| **Type of cost** | **Government** |
| --- | --- |
| Start-up | Planning |
|  | Planting material multiplication and dissemination |
|  | Sensitization, marketing, advocacy |
|  | Establishment of field schools |
|  | Demonstration days |
| Recurring | Planting material maintenance and dissemination |
|  | Ag extension and field schools |
|  | Marketing, advocacy, nutrition education |
|  | Nutrition surveillance |
|  | Management, overhead, and administration |

The annual recurring costs of biofortification are generally only a fraction of start-up costs (Lividini & Fiedler, 2015). After the start-up period, the SD tool default activities associated with operating a biofortification program are planting material maintenance and dissemination, the continued work of agricultural extensionists and the operation of field schools for quality control and continued support and education for farmers, and the continuation of marketing, advocacy, and nutrition education. We also assume the government will conduct follow-up national nutrition surveys approximately once every five years.

Assumptions about the rate of replacement (i.e., what percentage of potentially biofortifiable crop are actually biofortified) are built directly into the cost models at an annual time-step such that scale-up can occur over multiple years and/or biofortification programs operating “below capacity” can be reflected in the cost estimates. Similar to subnational fortification cost estimates, estimates of the subnational cost of biofortification are based on the total (undiscounted and discounted) national-level cost, allocated to each subnational unit according to consumption-based weights.

*(c) Supplementation cost models*

Supplements can be delivered via several different delivery platforms, including health campaigns (to deliver, e.g., high-dose vitamin A supplements to children under age 5), routine visits to health clinics, community health workers, etc. The general methods and structure of the cost models developed to estimate the cost of supplementation in the SD tool are similar across supplement delivery platforms, with some key differences.

The cost models for delivering supplements via any of the potential delivery platforms begins with estimating the cost per visit/contact in which the supplements are provided in terms of staff time and supplies. These estimates are based, first, on the amount of time (in minutes) for a nurse or other health system staff or volunteer to distribute the supplement via the specific delivery platform. Delivery platform-specific time estimates are provided in the OneHealth Input Assumptions Manual (<https://avenirhealth.org/software-onehealth.php>). For clinic-based delivery, supplement distribution that is assumed to happen as part of a routine clinic visit is assumed to take less time to distribute the supplement (just the extra time for supplement distribution) than for non-routine or “new” visits, where the total visit time is counted towards the cost of supplementation.

The annual health system personnel cost per person is then calculated as total time (in minutes) per visit/contact for supplement delivery multiplied by the assumed number of visits/contacts per year to delivery supplements (based on the assumptions presented in the OneHealth Input Assumptions Manual) and by the relevant health system staff salaries (calculated as a per-minute rate). This cost is then added to the cost of the supplements themselves (according to the UNICEF supply catalog) plus any relevant shipping, handling, and storage, to arrive at the total cost per member of the target population for activities directly related to the delivery of supplements.

The next set of costs are other facility-level recurrent and capital costs. These cost estimates are based on work done by the World Health Organization for their WHO-CHOICE tool and cost-effectiveness work to estimate country-specific costs for out-patient visits (see <https://www.who.int/choice/country/country_specific/en/> and Bollinger et al. (2017)). By isolating just other facility-level recurrent and capital costs from the total outpatient visit costs estimated by WHO, these serve as the per-visit estimates for other facility-level direct (e.g., non-consumables, facility-based training) and indirect (e.g., buildings, support personnel, maintenance, utilities, supervision/management at the facility level, etc.) cost associated with supplement programs provided at clinics or other health facilities. These costs are not applied to supplements delivered via health campaigns, community health workers, or other platforms outside of a health facility.

The final set of costs are program-level costs, which apply to all supplement delivery platforms, and include program-specific human resources, training, supervision, M&E, infrastructure, transport, communication, advocacy, etc. Again based on work done for WHO-CHOICE (see <https://www.who.int/choice/cost-effectiveness/inputs/price_non-traded/en/> and Johns, Baltussen & Hutubessy (2003), each component of these program costs are estimated as a percentage of total intervention costs.

In the SD tool, the OneHealth, LiST, and WHO-CHOICE time and cost estimates are used for the national-level default estimates in the SD tool. To estimate these costs at the subnational level, regional (or other subnational unit) adjustments are made to the national default estimates to reflect the impact of subnational differences in factors like population density, distance from ports of entry, and, if relevant, health system personnel salaries.

*(d) Intervention cost data sources*

Table S5 below summarizes, by type of intervention, potential secondary data sources and published and grey literature that might be tapped to inform the SD tool cost models and estimate intervention costs.

Table S5. Potential Data Sources to Inform Industry and Program Information and Unit Costs for Cost Model

| **Intervention** | **Industry and program information and unit costs** | **Potential secondary data source(s)** |
| --- | --- | --- |
| Fortification | Industry characteristics (size and scale of processing facilities) | - Trade association reports, e.g., World Grain (<https://www.world-grain.com/country_focus>), USDA annual grain and feed reports (<https://www.fas.usda.gov/commodities/grain-and-feed>) - United Nations Industrial Development Organization (UNIDO) databases (<https://www.unido.org/researchers/statistical-databases>) - Food fortification initiative (<http://www.ffinetwork.org/>) |
|  | Industry and government personnel wages | - International Labor Organization (<https://www.ilo.org/global/statistics-and-databases/lang--en/index.htm>) - Multipliers of published minimum wage rates |
|  | Availability of fortifiable food/condiment in the food supply | - FAO food balance sheets (<http://www.fao.org/faostat/en/#data/FBS>) - FEWS Net market reports (<https://fews.net/markets-and-trade>) |
|  | Percent of domestically produced potentially fortifiable food and percent imported | - FAO food balance sheets (<http://www.fao.org/faostat/en/#data/FBS>) - FEWS Net market reports (<https://fews.net/markets-and-trade>) |
|  | Fortification equipment costs | - Fiedler & Afidra (2010) - Fiedler & Macdonald (2009) - Alavi, Bugusu, Cramer et al. (2008) |
|  | Premix costs | - GAIN premix facility (<http://gpf.gainhealth.org/>) - Roks (2014) - Souganidis, Laillou, Leyvraz et al. (2013) - Fiedler & Macdonald (2009) |
|  | Quality control/monitoring equipment, chemical reagents, and labor costs | - UNICEF supply catalog (<https://supply.unicef.org/all-materials/nutrition.html>) - Fiedler & Afidra (2010) - Fiedler & Macdonald (2009) |
|  | Program performance | - Global Fortification Data Exchange (<https://fortificationdata.org/>) - FACT surveys (<https://www.gainhealth.org/resources/reports-and-publications/fortification-assessment-coverage-toolkit-fact>) |
| Biofortification | Availability of locally grown biofortifiable crop in the food supply and area under cultivation | - FAO food balance sheets (<http://www.fao.org/faostat/en/#data/FBS>) - IFPRI IMPACT data (<https://dataverse.harvard.edu/dataverse/impact>) |
|  | Subnational distribution of biofortifiable crop production | - Open Data for Africa (<http://dataportal.opendataforafrica.org/data/#topic=Agriculture>) |
|  | Wages | - International Labor Organization (<https://www.ilo.org/global/statistics-and-databases/lang--en/index.htm>) - Multipliers of published minimum wage rates |
|  | Planting material multiplication/maintenance and dissemination | - Meenakshi, Johnson, Manyong et al. (2010) - Lividini & Fiedler (2015) - Orange Flesh Sweet Potato Investment Guide (<http://www.sweetpotatoknowledge.org/wp-content/uploads/2016/02/OFSP-Investment-Implementation-Guide-Presentation_FINAL.pdf>) |
| Supplementation | Supplement costs (high-dose vitamin A capsules, iron-folic acid capsules, etc.) | UNICEF supply catalog (<https://supply.unicef.org/all-materials/pharmaceuticals/minerals-vitamins.html>)  UNICEF supply catalog (<https://supply.unicef.org/all-materials/pharmaceuticals/antianaemias.html>) |
|  | Health system staff (nurses, community health workers, etc.) salary | WHO-CHOICE (<https://www.who.int/choice/cost-effectiveness/en/>) via the Lives Saved Tool (LiST) costing module staff baseline estimates (<https://www.livessavedtool.org/>) |
|  | Health system staff working days per month and working hours per day | OneHealth Tool Input Assumptions Manual (<https://avenirhealth.org/software-onehealth.php>) |
|  | Personnel time (minutes) to distribute supplements per contact | OneHealth Tool Input Assumptions Manual (<https://avenirhealth.org/software-onehealth.php>) |
|  | Number of visits/contacts per year to deliver supplements | OneHealth Tool Input Assumptions Manual (<https://avenirhealth.org/software-onehealth.php>) |
|  | Other facility-level recurrent and capital costs | WHO-CHOICE (<https://www.who.int/choice/cost-effectiveness/en/>) via the Lives Saved Tool (LiST) costing module recurrent and capital cost estimates (<https://www.livessavedtool.org/>) |
|  | Program (above facility-level) costs as a percentage of total costs | WHO-CHOICE (<https://www.who.int/choice/cost-effectiveness/en/>) via the Lives Saved Tool (LiST) costing module recurrent and capital cost estimates (<https://www.livessavedtool.org/>) |

Table S6. Estimated apparent^1^ energy intakes

| **Children age 6-59 months** | | | | | |
| --- | --- | --- | --- | --- | --- |
|  |  | Mean intake | | Median intake | |
| Tool | Geography | kcal/d | SE | kcal/d | IQR |
| SD^2^ | National | 814 | 8 | 724 | (497, 1040) |
|  | Yaoundé/Douala | 860 | 16 | 804 | (590, 1055) |
|  | North | 755 | 16 | 752 | (483, 1175) |
|  | South | 839 | 11 | 787 | (547, 1093) |
| Full (all children)^3^ | National | 1069 | 12 | 1124 | (937, 1292 |
|  | Yaoundé/Douala | 1060 | 21 | 1186 | (986, 1340 |
|  | North | 1082 | 18 | 1079 | (853, 1336 |
|  | South | 1061 | 20 | 1127 | (981, 1252 |
| Full (non-breastfed children)^4^ | National | 1116 | 12 | 1187 | (1021, 1336) |
|  | Yaoundé/Douala | 1057 | 22 | 1219 | (1001, 1371) |
|  | North | 1158 | 20 | 1201 | (930, 1401) |
|  | South | 1105 | 19 | 1175 | (1064, 1280) |
| **Women of reproductive age** | | | | | |
|  |  | Mean intake | | Median intake | |
| Tool | Geography | kcal/d | SE | kcal/d | IQR |
| SD^2^ | National | 1864 | 13 | 1699 | (1187, 2349) |
|  | Yaoundé/Douala | 1959 | 26 | 1800 | (1325, 2435) |
|  | North | 1722 | 28 | 1772 | (1183, 2621) |
|  | South | 1909 | 18 | 1816 | (1306, 2472) |
| Full^5^ | National | 2235 | 17 | 2235 | (1810, 2707) |
|  | Yaoundé/Douala | 2031 | 27 | 2114 | (1681, 2562) |
|  | North | 2492 | 31 | 2471 | (2033, 2954) |
|  | South | 2148 | 26 | 2137 | (1736, 2573) |

IQR, interquartile range; SE, standard error.

^1^Because estimates from the MINIMOD-SD tool were based on household consumption and expenditure survey data, the term “apparent” is used to emphasize SD tool estimates were based on reported food acquisition and the assumptions that all food acquired during the recall period was consumed during the recall period without waste or food loss and that food was distributed to individual household members according to the age- and sex-specific energy requirements.

^2^Refers to estimates from the MINIMOD-SD tool based on household consumption and expenditure survey data.

^3^Refers to estimates from the full MINIMOD tool, based on 24-hour dietary recall data, estimated for the full sample of children age 6-59 months. Energy estimates from breastmilk consumption based on Brown, Dewey & Allen (1998).

^4^Refers to estimates from the full MINIMOD tool, based on 24-hour dietary recall data, estimated for the subsample of non-breastfed children age 6-59 months.

^5^Refers to estimates from the full MINIMOD tool based on 24-hour dietary recall data.

Table S7. Predicted effective coverage of individual and select combinations of vitamin A interventions: Children age 6-59 months

|  |  | **SD^1^** | | **Full (all children)^2^** | | **Full (non-breastfed children)^3^** | |
| --- | --- | --- | --- | --- | --- | --- | --- |
|  |  | % | SE | % | SE | % | SE |
| Refined oil fortification (12 mg/kg) | National | 13 | 0.6 | 18 | 4.1 | 22 | 5.1 |
|  | Yaoundé/Douala | 23 | 1.8 | 30 | 6.2 | 36 | 7.3 |
|  | North | 13 | 1.0 | 19 | 5.1 | 26 | 6.6 |
|  | South | 8 | 0.6 | 11 | 5.1 | 14 | 6.5 |
| Refined oil fortification (9 mg/kg) | National | 10 | 0.5 | 15 | 3.8 | 19 | 4.9 |
|  | Yaoundé/Douala | 20 | 1.8 | 27 | 7.0 | 32 | 8.2 |
|  | North | 10 | 0.8 | 15 | 4.0 | 21 | 5.2 |
|  | South | 6 | 0.5 | 10 | 5.0 | 12 | 6.3 |
| Wheat flour fortification (5.9 mg/kg) | National | 12 | 0.5 | 22 | 3.3 | 28 | 4.2 |
|  | Yaoundé/Douala | 32 | 2.0 | 39 | 4.7 | 46 | 5.4 |
|  | North | 10 | 0.7 | 20 | 5.1 | 27 | 6.5 |
|  | South | 6 | 0.5 | 17 | 5.3 | 21 | 6.6 |
| Bouillon cube fortification (80 mg/kg) | National | 10 | 0.5 | 19 | 4.8 | 25 | 6.0 |
|  | Yaoundé/Douala | 16 | 1.6 | 21 | 7.1 | 25 | 8.5 |
|  | North | 5 | 0.6 | 22 | 5.2 | 30 | 6.5 |
|  | South | 11 | 0.8 | 16 | 5.5 | 20 | 6.7 |
| Biofortified maize (1.25 mg RAE/kg) | National | 9 | 0.5 | 4 | 2.7 | 5 | 3.4 |
|  | Yaoundé/Douala | 6 | 0.9 | 2 | 3.4 | 3 | 4.1 |
|  | North | 13 | 1.0 | 9 | 2.2 | 12 | 2.8 |
|  | South | 8 | 0.6 | 0 | 5.8 | 1 | 7.1 |
| Biofortified maize (0.63 mg RAE/kg) | National | 4 | 0.3 | 1 | 2.1 | 1 | 2.7 |
|  | Yaoundé/Douala | 3 | 0.7 | 0 | 0.0 | 0 | 0.0 |
|  | North | 5 | 0.6 | 4 | 1.6 | 6 | 2.0 |
|  | South | 4 | 0.4 | 0 | 0.0 | 0 | 0.0 |
| High-dose VAS | National | 31 | 0.8 | 31 | 2.4 | 40 | 2.8 |
|  | Yaoundé/Douala | 41 | 2.1 | 35 | 3.7 | 41 | 4.5 |
|  | North | 22 | 1.3 | 39 | 6.6 | 52 | 8.4 |
|  | South | 33 | 1.2 | 23 | 5.8 | 29 | 7.0 |
| Refined oil fortification (12 mg/kg) + bouillon fortification (80 mg/kg) | National | 23 | 0.7 | 33 | 5.9 | 42 | 7.4 |
|  | Yaoundé/Douala | 36 | 2.0 | 40 | 5.3 | 47 | 6.5 |
|  | North | 22 | 1.2 | 40 | 10.5 | 55 | 13.5 |
|  | South | 19 | 1.0 | 24 | 6.0 | 29 | 7.4 |
| Refined oil fortification (12 mg/kg) + VAS | National | 49 | 0.9 | 38 | 3.0 | 49 | 3.5 |
|  | Yaoundé/Douala | 61 | 2.1 | 41 | 4.8 | 49 | 5.6 |
|  | North | 50 | 1.7 | 52 | 6.2 | 70 | 7.4 |
|  | South | 42 | 1.2 | 25 | 6.9 | 32 | 8.4 |
| Bouillon fortification (80 mg/kg) + VAS | National | 48 | 0.9 | 39 | 3.1 | 50 | 3.6 |
|  | Yaoundé/Douala | 57 | 2.1 | 40 | 4.4 | 48 | 5.4 |
|  | North | 47 | 1.7 | 53 | 5.8 | 72 | 6.7 |
|  | South | 46 | 1.3 | 26 | 7.2 | 33 | 8.7 |
| Refined oil fortification (12 mg/kg) + bouillon fortification (80 mg/kg) + VAS | National | 57 | 0.9 | 42 | 3.4 | 53 | 3.9 |
|  | Yaoundé/Douala | 66 | 2.0 | 42 | 5.1 | 50 | 6.0 |
|  | North | 63 | 1.7 | 58 | 5.6 | 79 | 6.4 |
|  | South | 49 | 1.3 | 27 | 7.6 | 34 | 9.2 |

SE, standard error.

^1^Refers to estimates from the MINIMOD-SD tool based on household consumption and expenditure survey data.

^2^Refers to estimates from the full MINIMOD tool and the full sample of children age 6-59 months, based on 24-hour dietary recall data, estimated for the full sample of children age 6-59 months.

^3^Refers to estimates from the full MINIMOD tool, based on 24-hour dietary recall data, estimated for the subsample of non-breastfed children age 6-59 months.

Table S8. Predicted effective coverage of individual and select combinations of folic acid interventions: WRA

|  |  | **SD^1^** | | **Full^2^** | |
| --- | --- | --- | --- | --- | --- |
|  |  | % | SE | % | SE |
| Fortified wheat flour (5 mg/kg) | National | 27 | 0.6 | 44 | 6.4 |
|  | Cities | 60 | 1.4 | 69 | 10.9 |
|  | North | 18 | 0.9 | 28 | 10.8 |
|  | South | 17 | 0.6 | 43 | 7.8 |
| Fortified bouillon (100 mg/kg) | National | 43 | 0.7 | 59 | 7.1 |
|  | Cities | 50 | 1.5 | 70 | 11.8 |
|  | North | 34 | 1.3 | 45 | 10.5 |
|  | South | 47 | 0.9 | 65 | 9.7 |
| Fortified wheat flour + fortified bouillon | National | 54 | 0.7 | 62 | 5.8 |
|  | Cities | 72 | 1.3 | 74 | 10.3 |
|  | North | 42 | 1.3 | 46 | 11.5 |
|  | South | 53 | 0.9 | 69 | 7.5 |

SE, standard error.

^1^Refers to estimates from the MINIMOD-SD tool based on household consumption and expenditure survey data.

^2^Refers to estimates from the full MINIMOD tool based on 24-hour dietary recall data.

Table S9. National and subnational nutrition benefits, costs, and cost-effectiveness of alternative vitamin A intervention programs over ten years (2020-2029)

|  |  | **Children effectively covered, ‘000s of child-years** | | **Total cost^3^, ‘000s of 2019 US$** | | **Cost per child-year effectively covered, 2019 US$** | |
| --- | --- | --- | --- | --- | --- | --- | --- |
|  |  | SD^1^ | Full^2^ | SD | Full | SD | Full |
| Refined oil fortification (12 mg/kg) | National | 5,121 | 6,262 | $7,349 | $7,306 | $1.44 | $1.17 |
|  | Yaoundé/Douala | 1,791 | 2,002 | $2,564 | $2,991 | $1.43 | $1.49 |
|  | North | 1,874 | 2,796 | $2,532 | $2,369 | $1.35 | $0.85 |
|  | South | 1,456 | 1,584 | $2,253 | $1,947 | $1.55 | $1.23 |
| Refined oil fortification (9 mg/kg) | National | 4,004 | 5,488 | $4,755 | $4,455 | $1.19 | $0.81 |
|  | Yaoundé/Douala | 1,710 | 1,831 | $1,659 | $1,824 | $0.97 | $1.00 |
|  | North | 1,250 | 2,282 | $1,638 | $1,445 | $1.31 | $0.63 |
|  | South | 1,044 | 1,452 | $1,458 | $1,187 | $1.40 | $0.82 |
| Wheat flour fortification (5.9 mg/kg) | National | 4,142 | 6,316 | $22,243 | $30,179 | $5.37 | $4.78 |
|  | Yaoundé/Douala | 2,203 | 2,064 | $10,452 | $11,782 | $4.74 | $5.71 |
|  | North | 1,015 | 2,367 | $6,536 | $7,127 | $6.44 | $3.01 |
|  | South | 924 | 1,968 | $5,255 | $11,270 | $5.69 | $5.73 |
| Bouillon cube fortification (80 mg/kg) | National | 2,857 | 4,885 | $8,391 | $9,802 | $2.94 | $2.01 |
|  | Yaoundé/Douala | 1,011 | 958 | $1,987 | $2,280 | $1.97 | $2.38 |
|  | North | 490 | 2,252 | $2,182 | $3,581 | $4.45 | $1.59 |
|  | South | 1,356 | 1,708 | $4,222 | $3,940 | $3.11 | $2.31 |
| Biofortified maize (1.25 mg RAE/kg) | National | 2,621 | 1,057 | $4,460 | $1,327 | $1.70 | $1.26 |
|  | Yaoundé/Douala | 357 | 105 | $467 | $139 | $1.31 | $1.33 |
|  | North | 1,173 | 900 | $1,851 | $720 | $1.58 | $0.80 |
|  | South | 1,091 | 46 | $2,142 | $468 | $1.96 | $10.07 |
| Biofortified maize (0.63 mg RAE/kg) | National | 1,157 | 289 | $3,904 | $1,327 | $3.38 | $4.59 |
|  | Yaoundé/Douala | 180 | 0 | $405 | $139 | $2.25 | n/a |
|  | North | 422 | 460 | $1,598 | $720 | $3.78 | $1.56 |
|  | South | 555 | 0 | $1,901 | $468 | $3.43 | n/a |
| High-dose VAS | National | 12,223 | 11,528 | $32,074 | $30,401 | $2.62 | $2.64 |
|  | Yaoundé/Douala | 3,549 | 2,351 | $6,085 | $5,820 | $1.71 | $2.48 |
|  | North | 2,824 | 5,818 | $10,790 | $10,507 | $3.82 | $1.81 |
|  | South | 5,851 | 3,502 | $15,199 | $14,074 | $2.60 | $4.02 |
| Refined oil fortification (12 mg/kg) + bouillon fortification (80 mg/kg) | National | 7,812 | 10,301 | $15,740 | $17,108 | $2.01 | $1.66 |
|  | Yaoundé/Douala | 2,701 | 2,463 | $4,551 | $5,271 | $1.69 | $2.14 |
|  | North | 2,396 | 5,070 | $4,714 | $5,950 | $1.97 | $1.17 |
|  | South | 2,716 | 2,923 | $6,474 | $5,887 | $2.38 | $2.01 |
| Refined oil fortification (12 mg/kg) + VAS | National | 19,164 | 13,643 | $39,422 | $37,707 | $2.06 | $2.76 |
|  | Yaoundé/Douala | 5,214 | 2,700 | $8,649 | $8,811 | $1.66 | $3.26 |
|  | North | 6,375 | 7,420 | $13,322 | $12,876 | $2.09 | $1.74 |
|  | South | 7,575 | 3,730 | $17,452 | $16,021 | $2.30 | $4.30 |
| Bouillon fortification (80 mg/kg) + VAS | National | 17,170 | 13,600 | $40,465 | $40,203 | $2.36 | $2.96 |
|  | Yaoundé/Douala | 4,537 | 2,611 | $8,073 | $8,101 | $1.78 | $3.10 |
|  | North | 5,099 | 7,402 | $12,972 | $14,088 | $2.54 | $1.90 |
|  | South | 7,533 | 3,797 | $19,420 | $18,014 | $2.58 | $4.74 |
| Refined oil fortification (12 mg/kg) + bouillon fortification (80 mg/kg) + VAS | National | 21,478 | 14,972 | $47,814 | $47,509 | $2.23 | $3.17 |
|  | Yaoundé/Douala | 5,496 | 2,831 | $10,637 | $11,091 | $1.94 | $3.92 |
|  | North | 7,551 | 8,437 | $15,504 | $16,457 | $2.05 | $1.95 |
|  | South | 8,431 | 3,964 | $21,673 | $19,961 | $2.57 | $5.04 |

^1^Refers to estimates from the MINIMOD-SD tool based on household consumption and expenditure survey data.

^2^Refers to estimates from the full MINIMOD tool based on 24-hour dietary recall data.

^3^The movement of food via markets means that large-scale food fortification and biofortification interventions are not targetable in the sense that they could implemented in certain regions but not others. Subnational costs were therefore based on consumption-based weights. That is, the total cost of fortification and biofortification interventions was disaggregated by macro-region in proportion to each macro-region’s share of total consumption of the food vehicle.

Table S10. National and subnational nutrition impacts, costs, and cost-effectiveness of alternative folic acid intervention programs over ten years (2020-2029)

|  |  | **WRA-years effectively covered, ‘000s of child-years** | | **Total cost^3^, ‘000s of 2019 US$** | | **Cost per WRA-year effectively covered, 2019 US$** | |
| --- | --- | --- | --- | --- | --- | --- | --- |
|  |  | SD^1^ | Full^2^ | SD | Full | SD | Full |
| Fortified wheat flour (5 mg/kg) | National | 17,984 | 25,109 | $15,068 | $15,400 | $0.84 | $0.61 |
|  | Yaoundé/Douala | 8,883 | 10,559 | $7,080 | $6,012 | $0.80 | $0.57 |
|  | North | 4,012 | 5,180 | $4,428 | $3,637 | $1.10 | $0.70 |
|  | South | 5,090 | 10,181 | $3,560 | $5,751 | $0.70 | $0.56 |
| Fortified bouillon (100 mg/kg) | National | 23,209 | 30,436 | $2,650 | $3,313 | $0.11 | $0.11 |
|  | Yaoundé/Douala | 5,855 | 9,402 | $628 | $771 | $0.11 | $0.08 |
|  | North | 5,855 | 7,483 | $689 | $1,211 | $0.12 | $0.16 |
|  | South | 11,498 | 13,679 | $1,333 | $1,332 | $0.12 | $0.10 |
| Fortified wheat flour + fortified bouillon | National | 32,293 | 34,783 | $17,718 | $18,714 | $0.55 | $0.54 |
|  | Yaoundé/Douala | 10,214 | 11,188 | $7,708 | $6,783 | $0.75 | $0.61 |
|  | North | 8,166 | 8,226 | $5,117 | $4,847 | $0.63 | $0.59 |
|  | South | 13,913 | 15,617 | $4,893 | $7,083 | $0.35 | $0.45 |

^1^Refers to estimates from the MINIMOD-SD tool based on household consumption and expenditure survey data.

^2^Refers to estimates from the full MINIMOD tool based on 24-hour dietary recall data.

^3^The movement of food via markets means that large-scale food fortification and biofortification interventions are not targetable in the sense that they could implemented in certain regions but not others. Subnational costs were therefore based on consumption-based weights. That is, the total cost of fortification and biofortification interventions was disaggregated by macro-region in proportion to each macro-region’s share of total consumption of the food vehicle.

References

(2006). Guidelines on food fortification with micronutrients. (eds L.H. Allen, B. de Benoist, O. Dary & R. Hurrell). The World Health Organization and the Food and Agricultural Organization of the United Nations, http://www.who.int/nutrition/publications/guide_food_fortification_micronutrients.pdf.

Alavi S., Bugusu B., Cramer G., Dary O., Lee T.-C., Martin L., . . . Wailes E. (2008). Rice fortification in developing countries: A critical review of the technical and economic feasibility. A2Z Project Washington, D.C., https://www.spring-nutrition.org/sites/default/files/a2z_materials/508-food-rice-fortification-report-with-annexes-final.pdf.

Bollinger L.A., Sanders R., Winfrey W. & Adesina A. (2017). Lives saved tool (list) costing: A module to examine costs and prioritize interventions. *BMC Public Health,* 17, 21-28.

Brown K., Dewey K. & Allen L. (1998). *Complementary feeding of young children in developing countries: A review of current scientific knowledge.* Geneva, World Health Organization.

Fiedler J.L. & Afidra R. (2010). Vitamin A fortification in Uganda: Comparing the feasibility, coverage, costs, and cost-effectiveness of fortifying vegetable oil and sugar. *Food and Nutrition Bulletin,* 31, 193-205.

Fiedler J.L. & Macdonald B. (2009). A strategic approach to the unfinished fortification agenda: Feasibility, costs, and cost-effectiveness analysis of fortification programs in 48 countries. *Food and Nutrition Bulletin,* 30, 283-316.

Global Fortification Data Exchange (2020). Dashboard: Cameroon fortificatoin. https://fortificationdata.org/country-fortification-dashboard/?alpha3_code=CMR&lang=en.

Institute of Medicine (1998). *Dietary reference intakes for thiamin, riboflavin, niacin, vitamin b6, folate, vitamin b12, pantothenic acid, biotin, and choline.* National Academies Press, Washington, D.C.

Institute of Medicine (2001). *Dietary reference intakes for Vitamin A, vitamin k, arsenic, boron, chromium, copper, iodine, iron, manganese, molybdenum, nickel, silicon, vanadium, and zinc.* National Academies Press, Washington, D.C.

Johns B., Baltussen R. & Hutubessy R. (2003). Programme costs in the economic evaluation of health interventions. *Cost Effectiveness and Resource Allocation,* 1, 1.

Lividini K. & Fiedler J.L. (2015). Assessing the promise of biofortification: A case study of high provitamin a maize in Zambia. *Food Policy,* 54, 65-77.

Mark H.E., Assiene J.G., Luo H., Nankap M., Ndjebayi A., Ngnie-Teta I., . . . Engle-Stone R. (2019). Monitoring of the national oil and wheat flour fortification program in Cameroon using a program impact pathway approach. *Current developments in nutrition,* 3, nzz076-nzz076.

Meenakshi J.V., Johnson N.L., Manyong V.M., DeGroote H., Javelosa J., Yanggen D.R., . . . Meng E. (2010). How cost-effective is biofortification in combating micronutrient malnutrition? An ex ante assessment. *World Development,* 38, 64-75.

Republique du Cameroun Institut National de la Statistique (2007). Troisieme enquete aupres des menages (ecam3): Document de methodologie generale. Institut National du Statistique, Yaounde, Cameroon.

Roks E. (2014). Review of the cost components of introducing industrially fortified rice. 1324, 82-91.

Souganidis E., Laillou A., Leyvraz M. & Moench-Pfanner R. (2013). A comparison of retinyl palmitate and red palm oil β-carotene as strategies to address Vitamin A deficiency. 5, 3257-3271.
